# A multi-scale model for determining the effects of pathophysiology and metabolic disorders on tumor growth

**DOI:** 10.1038/s41598-020-59658-0

**Published:** 2020-02-20

**Authors:** Mohammad Reza Nikmaneshi, Bahar Firoozabadi, Aliasghar Mozafari, Lance L. Munn

**Affiliations:** 1 Edwin L. Steele Laboratories, Department of Radiation Oncology, Massachusetts General Hospital, Harvard Medical School, Boston, MA 02114 USA; 2 Department of Mechanical Engineering, Sharif University of Technology, Tehran, Iran

**Keywords:** Mechanical engineering, Computational science, Fluid dynamics, Biomedical engineering, Chemical engineering

## Abstract

The search for efficient chemotherapy drugs and other anti-cancer treatments would benefit from a deeper understanding of the tumor microenvironment (TME) and its role in tumor progression. Because *in vivo* experimental methods are unable to isolate or control individual factors of the TME and *in vitro* models often do not include all the contributing factors, some questions are best addressed with systems biology mathematical models. In this work, we present a new fully-coupled, agent-based, multi-scale mathematical model of tumor growth, angiogenesis and metabolism that includes important aspects of the TME spanning subcellular-, cellular- and tissue-level scales. The mathematical model is computationally implemented for a three-dimensional TME, and a double hybrid continuous-discrete (DHCD) method is applied to solve the governing equations. The model recapitulates the distinct morphological and metabolic stages of a solid tumor, starting with an avascular tumor and progressing through angiogenesis and vascularized tumor growth. To examine the robustness of the model, we simulated normal and abnormal blood conditions, including hyperglycemia/hypoglycemia, hyperoxemia/hypoxemia, and hypercarbia/hypocarbia – conditions common in cancer patients. The results demonstrate that tumor progression is accelerated by hyperoxemia, hyperglycemia and hypercarbia but inhibited by hypoxemia and hypoglycemia; hypocarbia had no appreciable effect. Because of the importance of interstitial fluid flow in tumor physiology, we also examined the effects of hypo- or hypertension, and the impact of decreased hydraulic conductivity common in desmoplastic tumors. The simulations show that chemotherapy-increased blood pressure, or reduction of interstitial hydraulic conductivity increase tumor growth rate and contribute to tumor malignancy.

## Introduction

The tumor microenvironment (TME) is an anatomically and physiologically complex environment, with important processes at multiple size scales controlled by diverse biomechanical and biochemical signals, all contained within an extracellular matrix (ECM) – rich, abnormal stroma^1^. Because of the inherent complexity of the TME, it is difficult to determine the important components and processes responsible for tumor growth, angiogenesis and metastasis using currently available experimental models *in vitro* or *in vivo*. Consequently, the treatments developed using these experimental models often prove less effective in human patients^2^. Animal models, despite including representative TME abnormalities, are not easily amenable to the isolation or manipulation of individual biological factors to determine unequivocal causation^3^. Although micro fabricated *in vitro* models and engineered tissues are able to recapitulate some aspects of the TME such as angiogenesis^4–10^, tumor cell invasion and metastasis^10–13^, avascular and vascular tumor-growth^14–16^, they cannot recapitulate all the complexities of the TME seen in patient tumors. Nevertheless, it is possible to reproduce important complexities of the TME using systems biology models, which allow easy manipulation of individual environmental factors to determine how each impacts tumor progression^17–25^.

Solid tumor growth and angiogenesis are induced by autocrine and paracrine biochemical pathways that modulate the behavior of tumor cells (TCs) and endothelial cells (ECs)^26^. Tumor cells can become necrotic, quiescent, migratory or proliferative in response to these signals, and these processes determine the dynamics and morphology of a solid tumor growing within its TME^20^. For example, cellular respiration (CR), chemotaxis/haptotaxis, and ECM production/degradation are all important processes within the TME that affect tumor biology^5,27–29^, but are difficult to study using experimental systems. The biochemical signaling pathways are also directly influenced by physical abnormalities in the TME. For example, high interstitial fluid pressure (IFP) within tumors causes increased interstitial fluid flow (IFF) at the tumor boundary, which affects biochemical transport and distribution within the tumor^24^.

Once a primary solid tumor exceeds a threshold volume (~1 mm)^30^, its central cells become starved because the outer surrounding cells deplete the nutrients; in response, the central cells secrete a variety of morphogenic and chemotactic growth factors^31^ such as vascular endothelial growth factor (VEGF), angiopoietin-1, and -2 (Ang-1 and Ang-2)^4,32^. VEGF binding to its receptor, VEGFR-2, on ECs induces differentiation of ECs and formation of tip endothelial cells (tECs) that extend from the existing vessel wall to create sprouts in the initial stage of angiogenesis^33,34^. tECs lead the migration of vessel sprouts into the surrounding tissue, biased toward areas of high VEGF concentration (chemotaxis), and traveling through regions where the ECM supports cell-matrix interactions (haptotaxis)^35,36^. During migration, tECs secrete MMPs, which proteolytically degrade structural components of the ECM^5,37^. Following behind the tECs are the stalk endothelial cells (sECs), which are induced by NOTCH signaling to proliferate and form lumens^5,33,34^. As the migrating vessels meet and form connections, this process of VEGF-induced angiogenesis creates a complex network of immature neo-vessels that supports blood flow into the tumor. Another cytokine, Ang-1 causes maturation and stabilization of neo-vessels through binding to Tie-2 of ECs and, in contrast, Ang-2 promotes death of ECs and destabilization of neo-vessels by competing with Ang-1 for Tie-2 binding sites^38–40^.

The literature is replete with models of avascular tumor growth (ATG)^29,41^, vascular tumor growth (VTG)^17,20^, and of the transition from avascular to vascular growth (ATG-to-VTG)^19,21,22,42–44^, all of which can be useful for examining tumor growth and morphology. Governed by the coordinated cellular dynamics of tECs and sECs, tumor-induced angiogenesis involves sprouting, branching, anastomosis, rupturing, and remodeling of neo-vessels^32,45^. Mathematical models that include angiogenesis can be divided into three classifications: (i) conventional, continuous models that calculate EC density without explicit representation of the structure of the neo-vessels or network^24,44,46–49^, (ii) discrete models that predict EC migration and the creation of neo-vessels based on constant predefined, probabilistic motion^18,50,51^, and (iii) recent hybrid, continuous-discrete models that trace the EC pathways using variable agent-based motion probabilities^45,52–54^. As such, the mathematical models of avascular tumor-growth (ATG)^29,41^, vascular tumor growth (VTG)^17,20^, and ATG-to-VTG^19,21,22,42–44^ utilize these three computational approaches to determine tumor morphology. The most comprehensive models for recapitulating the TME are ATG-to-VTG models with angiogenesis, which have been successfully developed for 2-D^21,42,43^ and 3-D^19,22,55,56^ domains. Table 1 summarizes previous three-dimensional models of tumor growth and angiogenesis and the relevant aspects of the current work.

**Table 1:** Three-dimensional Mathematical models of the tumor microenvironment and angiogenesis.

Ref	Domain		Tumor Growth			Model Formu-lation	Subcellular (Molecular level)	Cellular Level		Angiogenesis Vessels					Fluid Dynamics			Solid Mech-anics	Notes
	Tumor Tissue	Blood Vessels	Avascular Growth	Angio-genesis	Vascular Growth			Tumor Cells	Endo-thelial Cells	Lumeno-genesis	Vessel Adap-tation	Vessel Defor-mation	Bran-ching	Disru-ption	Hemo-rheology	Hemo-dyna-mics	Inter-stitial Fluid Flow		
3D models																			
Das, *et al*.^45^	−	+	−	+	−	**HCD**	**FN, MMP, VEGF**	−	+**,a**	−	−	−	+**,b**	−	−	−	−	−	^a^TAF gradient−induced chemotaxis and fibronectin gradient−induced haptotaxis; ^b^Stochastic
Welter and Rieger^52^	+	+	−	+	+	**Discrete/HCD,a**	**O**_**2**_**, FN, VEGF**	+**,b**	+**,c**	−	−	−	+**,d**	+**,e**	+**,f**	+	+	−	^a^Discrete model for endothelial cells; HCD model for tumor cells: probabilities based on O_2;_^b^Cell phenotype depends on O_2_; ^c^Stochastic and continuous processes for angiogenesis; ^d^Based on a threshold length for sprouts; ^e^Based on wall shear−stress (WSS); ^f^Viscosity depends on hematocrit, vessel diameter
Milde, *et al*.^54^	−	+	−	+	−	**HCD**	**FN, MMP, VEGF**	−	+**,a**	−	−	−	+**,b**	−	−	−	−	−	^a^TAF gradient−induced chemotaxis and fibronectin gradient−induced haptotaxis; ^b^Based on sprout age
Wang, *et al*.^41^	+	−	+	−	−	**Discrete**	**O**_**2**_**, glucose, EGF, TGF**	+**,a**	−	−	−	−	−	−	−	−	−	−	^a^Cell phenotype depends on glucose, EGF and TGF
Cai, *et al*.^20^	+	+	+	+	+	**HCD**	**O**_**2**_**, FN, MMP, VEGF**	+**,a**	+**,b**	−	−	+	+**,c**	+**,d**	+**,e**	+	+	−	^a^Cell phenotype depends on O_2_; ^b^TAF gradient−induced chemotaxis and fibronectin gradient−induced haptotaxis; ^c^Based on sprout age; ^d^Based on WSS; ^e^Viscosity depends on hematocrit and vessel diameter
Tang, *et al*.^19^	+	**+**	**+**	**+**	**+**	**HCD**	**O**_**2**_**, CO**_**2**_**, TAF**	**+,a**	**+,b**	−	−	−	**+,c**	−	−	−	−	**+,d**	^a^O_2_ and CO_2_ affect viability and cellular energy; ^b^TAF gradient−induced chemotaxis for sprouting, TAF−induced branching, Transvascular pressure for angiogenesis; ^c^Based on VEGF concentration; ^d^A Gaussian function to model tumor cell−induced solid pressure
Vavourakis, *et al*.^22^	**+**	**+**	**+**	**+**	**+**	**Continuous/HCD,a**	**O**_**2**_**, FN, MMP, TAF**	−	**+.b**	−	**+,c**	**+**	**+,d**	**+,e**	−	**+**	**+**	**+,f**	^a^Continuous model for tumor growth; HCD model for angiogenesis: probabilities based on TAF; ^b^TAF gradient−induced chemotaxis and fibronectin gradient−induced haptotaxis; ^c^Remodeling depends on WSS; ^d^Stochastic; ^e^Based on flow velocity; ^f^Navier−Cauchy equation
Cai, *et al*.^55^	**+**	**+**	**+**	**+**	**+**	**HCD**	**O**_**2**_**, FN, MMP, VEGF**	**+,a**	**+,b**	−	−	**+**	−	−	−	**+**	**+**	−	^a^Phenotype depends on O_2_; ^b^TAF gradient-induced chemotaxis and fibronectin gradient-induced haptotaxis
Shirinifard, *et al*.^56^	**+**	**+**	**+**	**+**	**+**	**HCD**		**+,a**	**+**	−	−	−	−	**+,b**	−	−	−	−	^a^Cell phenotype depends on O_2_; ^b^Based on length of vessel segment
Norton and Popel^51^	−	**+**	−	**+**	−	**Discrete**	**Predefined VEGF profile**	−	−	−	−	−	−	−	−	−	−	−	
Present model	**+**	**+**	**+**	**+**	**+**	**DHCD, a**	**O**_**2**_**, glucose, CO**_**2**_**, FN, MMP, VEGFR-2, Ang-1, Ang-2, Tie-2**	**+,b**	**+,c**	**+,d**	**+,e**	**+**	**+,f**	**+,g**	**+,h**	**+**	**+**	**+,i**	^a^Probabilities based on chemo-, hapto-taxis and fluid pressure/solid stress^; b^Cellular respiration-based vitality and ATP-based energy determine phenotypes; ^c^VEGF gradient-induced chemotaxis/fibronectin gradient-induced haptotaxis for sprouting; Ang-1 and Ang-2 competition for lumenogenesis and maturation of stalk cells; VEGF-induced differentiation of stalk to tip cells for branching; Transvacuolar pressure affects tip cell migration; ^d^Stalk cell states based on VEGF, Ang-1 and Ang-2; ^e^Depends on WSS; Intravascular pressure; VEGF (VEGF-dependent susceptibility to adaptation); Metabolic mechanism based on hematocrit; ^f^Based on VEGF concentration /stalk cells differentiate to tip cells; ^g^Based on WSS and VEGF; ^h^Vessel diameter and hematocrit affect viscosity; bifurcations affect hematocrit; ^i^A Gaussian function imposes tumor cell-induced solid stress

**Figure 1:**
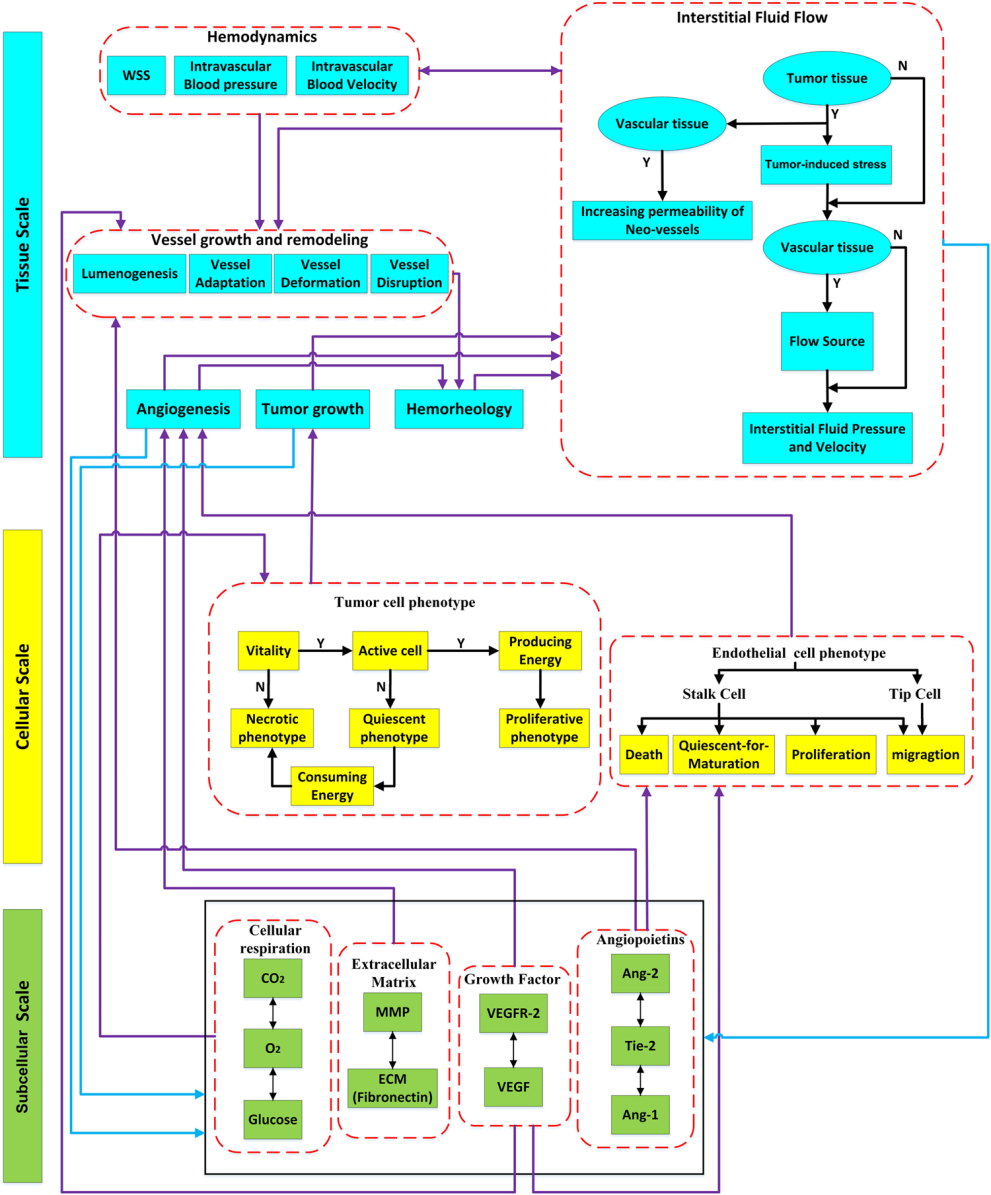
The overall algorithm for simulating a three-dimensional TME with subcellular, cellular, and tissue size scales.

In this work, we developed a comprehensive, fully-coupled multi-scale mathematical model that recapitulates a three-dimensional TME to better understand the transition from avascular growth to angiogenesis and VTG. As shown in Fig. 1, our model includes subcellular-, cellular-, and tissue-level size scales. The subcellular scale is the basic scale of the model, and consists of the biochemical agents: ECM (fibronectin), MMP, VEGF and VEGFR-2, Ang-1 & Ang-2 and their common receptor Tie-2, and CR-agents. At the cellular scale, proliferating, quiescent, and necrotic phenotypes of TCs are determined through a novel CR-based method. Unlike many previous studies that consider oxygen as the only factor determining cell viability, we include multiple CR-agents – oxygen, glucose and carbon dioxide – to determine cellular activities and phenotypes. For the ECs involved in angiogenesis, we use agent-based modeling to determine proliferation, quiescence persistence, death and migration of the cells. At the tissue scale, solid tumor growth and blood vasculature are implemented by a new double hybrid continues-discrete (DHCD) model with the highest degree-of-freedom (DOF) for movement of TCs and tECs in three-dimensional space. At the tissue level, we also model vessel remodeling, which is determined by biochemical and mechanobiological signals from blood shear stress with accurate hemodynamics and hemorheology^57^. We also consider blood pressure explicitly, and calculate interstitial fluid flow, which influences the transport of soluble species.

## Methods

The overall algorithm of the present multi-scale TME model is shown in Fig. 1. Subcellular, cellular and tissue scales and their components are represented by green, yellow, and blue colors, respectively. All model compartments and their communications were designed to facilitate calibration of model parameters based on experimental observations for all stages of the simulations (avascular tumor growth, angiogenesis and vascular tumor growth).

### Subcellular scale: biochemical agents

According to the CR process, glucose, oxygen and carbon-dioxide influence the production of mitochondrial-synthesized adenosine-triphosphate (ATP), which is the basis of cellular energy^28,58^. Here, we use a set of reaction-convection-diffusion mathematical equations to model absorption-distribution-metabolite-excretion (ADME) of each CR-agent. The ADME equations for concentrations of oxygen, ${c}_{{o}_{2}}$, glucose, ${c}_{g}$, and carbon-dioxide, ${c}_{c{o}_{2}}$, are respectively written in Eqs. 1–3:

1 $$\frac{\partial {c}_{{o}_{2}}}{\partial t}+{\boldsymbol{\nabla }}.({{\boldsymbol{u}}}_{{\boldsymbol{ins}}}{c}_{{o}_{2}})={D}_{{o}_{2}}{{\boldsymbol{\nabla }}}^{2}{c}_{{o}_{2}}-\mathop{\underbrace{\,{C}_{{o}_{2}}{L}_{TC}}}\limits_{consumption\,}+\mathop{\underbrace{{E}_{{o}_{2}}{L}_{EC}}}\limits_{perfusion\,}$$

2 $$\frac{\partial {c}_{g}}{\partial t}+{\boldsymbol{\nabla }}.({{\boldsymbol{u}}}_{{\boldsymbol{ins}}}\,{c}_{g})={D}_{g}{{\boldsymbol{\nabla }}}^{2}{c}_{g}-\mathop{\underbrace{\,{C}_{g}{L}_{TC}}}\limits_{consumption\,}+\mathop{\underbrace{{E}_{{\rm{g}}}{L}_{EC}}}\limits_{perfusion\,}$$

3 $$\frac{\partial {c}_{c{o}_{2}}}{\partial t}+{\boldsymbol{\nabla }}.({{\boldsymbol{u}}}_{{\boldsymbol{ins}}}\,{c}_{c{o}_{2}})={D}_{c{o}_{2}}{{\boldsymbol{\nabla }}}^{2}{c}_{c{o}_{2}}+\mathop{\underbrace{\,{R}_{c{o}_{2}}{L}_{TC}}}\limits_{production\,}-\mathop{\underbrace{{E}_{c{o}_{2}}{L}_{EC}}}\limits_{perfusion\,}$$

*D* is the diffusion coefficient of each respective agent, ***u***_***ins***_ is the interstitial fluid flow (IFF) velocity, *C* and *R* are consumption and production rates, respectively, *E* is the perfusion rate, and *L* indicates the location of ECs (*L*_*EC*_), and TCs (*L*_*TC*_). IFF and convection of soluble agents is considered throughout the domain. This is especially relevant near the vessels, where the Peclet number can be as high as ~1. Glucose and oxygen diffuse and are advected in the tissue from the source blood vessels. Carbon dioxide is produced in the tissue and taken up by the blood vessels, which are represented as sinks in the model. Assumed to be a function of cellular vitality (*ϑ*), consumption of glucose and oxygen, and production of carbon-dioxide by TCs are related by the stoichiometry of the CR-reaction according to:

4 $$\begin{array}{l}CR-reaction:{C}_{6}{H}_{12}{O}_{6}+6{O}_{2}\to 6C{O}_{2}+6{H}_{2}O+ATP\\ 6\,\times {C}_{g}(\vartheta )={C}_{{o}_{2}}(\vartheta )={R}_{c{o}_{2}}(\vartheta )={\gamma }_{0}\vartheta ({c}_{g},{c}_{{o}_{2}},{c}_{c{o}_{2}})\end{array}$$

*γ*_0_ is the maximum consumption or production rate of the CR agent. The transport of oxygen and carbon-dioxide – two hydrophobic molecules – is calculated using a simplified perfusion model based on transvascular pressure, *p* (the difference between intravascular blood pressure, *p*_*lum*_, and interstitial fluid pressure, *p*_*ins*_) and vessel diameter, *d*_*v*_^19^. However, transport of glucose, a hydrophilic polar molecule, is more complex^59^. In addition to transvascular pressure and vessel diameter, efflux from blood and influx to the TCs can affect the glucose transfer^60^. Mammalian TCs have transmembrane symporter proteins that automatically intake glucose without the need for ATP consumption^61^. Therefore, we include only a Michaelis-Menten (M-M) model to determine glucose efflux from the blood to TCs. Finally, the convection components of oxygen, glucose and carbon-dioxide transvascular transport are derived through Eq. 5a–c, respectively:

5a $${E}_{{o}_{2}}({d}_{v},p)={f}_{{o}_{2}}\frac{{d}_{v}}{{d}_{c}}(\frac{p}{{p}_{lum}})$$

5b $${E}_{g}({d}_{v},p)=({f}_{g}\frac{{c}_{g}}{{c}_{g}+k{m}_{g}})\frac{{d}_{v}}{{d}_{c}}(\,\frac{p}{{p}_{lum}})$$

5c $${E}_{c{o}_{2}}({d}_{v},p)={f}_{c{o}_{2}}\frac{{d}_{v}}{{d}_{c}}(\frac{p}{{p}_{lum}})$$

${f}_{{o}_{2}}$ is the rate constant for oxygen transport into tissue, ${f}_{g}$ and $k{m}_{g}$are the maximum rate and M-M constant of glucose transport from blood, ${f}_{c{o}_{2}}$ is the transport rate of carbon-dioxide, and ${d}_{c}$is the characteristic diameter of a neo-vessel.

TCs exposed to oxygen concentration below a threshold level, ${c}_{{o}_{2}}^{ch}$, secrete VEGF to stimulate ECs of nearby vessels to sprout. Once ECs become associated with the tumor tissue, their VEGFR-2 receptors are expressed^38^. The ADME model of VEGF coupled with VEGFR-2 is given in Eqs. 6–8:

6 $$\begin{array}{ccc}\frac{{\rm{\partial }}{c}_{v}}{{\rm{\partial }}t}+{\boldsymbol{\nabla }}.({{\boldsymbol{u}}}_{{\boldsymbol{i}}{\boldsymbol{n}}{\boldsymbol{s}}}\,{c}_{v}) & = & {D}_{v}\,{{\boldsymbol{\nabla }}}^{2}{c}_{v}+\mathop{\underbrace{\,H({c}_{{o}_{2}}^{ch}-{c}_{{o}_{2}})(1-\frac{{c}_{{o}_{2}}}{{c}_{{o}_{2}}^{ch}}){R}_{v}\,{L}_{TC}}}\limits_{production\,}-\mathop{\underbrace{{f}_{v}\,\frac{{d}_{v}}{{d}_{c}}\,{L}_{EC}}}\limits_{perfusion\,}\\  &  & \,-\mathop{\underbrace{{k}_{v}^{+}\,{r}_{v}^{f}\,{c}_{v}}}\limits_{bound\,to\,free\,VEGFR-2\,}+\mathop{\underbrace{{k}_{v}^{-}\,{r}_{v}^{a}}}\limits_{unbound\,from\,active\,VEGFR-2}-\mathop{\underbrace{{\varepsilon }_{v}{c}_{v}}}\limits_{excretion}\end{array}$$

7 $$\frac{d{r}_{v}^{f}}{dt}=-{k}_{v}^{+}{r}_{v}^{f}{c}_{v}+{k}_{v}^{-}\,{r}_{v}^{a}$$

8 $$\frac{d{r}_{v}^{a}}{dt}={k}_{v}^{+}{r}_{v}^{f}{c}_{v}-{k}_{v}^{-}{r}_{v}^{a}$$

${R}_{v}$ is the rate of production of VEGF by TCs, ${f}_{v}$ is the VEGF transport rate between tissue and vessels,$\,{k}_{v}^{+}$ is the binding rate of VEGF to VEGFR-2,$\,{k}_{v}^{-}$ is the dissociation rate of VEGF from VEGFR-2, ${\varepsilon }_{v}$ is the natural excretion rate of VEGF, ${r}_{v}^{f}$ is the concentration of free-VEGFR-2, and ${r}_{v}^{a}$ is the concentration of active-VEGFR-2 bound to VEGF. As such, $H({c}_{{o}_{2}}^{ch}-{c}_{{o}_{2}})$ is a Heaviside function to activate VEGF secretion when oxygen concentration, ${c}_{{o}_{2}}$, falls below the characteristic value, ${c}_{{o}_{2}}^{ch}$.

Ang-1 is secreted by ECs in the vicinity of both healthy and tumor tissue, and Ang-2 is secreted by ECs associated with tumor tissue^38–40^. The ADMEs of Ang-1, ${c}_{a1}$, and Ang-2, ${c}_{a2}$, coupled with Tie-2 are mathematically modeled through Eqs. 9–13. ${R}_{a1}$ and ${R}_{a2}$ are respectively the secretion rates of Ang-1 by ECs and of Ang-2 by ECs associated with tumor tissue. ${e}_{0}$ is the characteristic concentration of ECs in each blood vessel, ${K}_{a}$ is the carrying capacity coefficient of angiopoietins, ${k}_{a1}^{+}$ and ${k}_{a1}^{-}$ are respectively Ang-1 binding rate to and unbinding rate from Tie-2, ${k}_{a2}^{+}$ and ${k}_{a2}^{-}$ are respectively Ang-2 binding rate to and unbinding rate from Tie-2, ${\varepsilon }_{a1}\,$ and ${\varepsilon }_{a2}\,$ are the natural excretion rates of Ang-1 and Ang-2, respectively. ${r}_{a}^{f}$ is the concentration of free Tie-2, ${r}_{a1}^{a}$ and ${r}_{a2}^{a}$ are concentrations of active Tie-2 bound to Ang-1 and Ang-2, respectively.

9 $$\begin{array}{c}\begin{array}{rcl}\frac{\partial {c}_{a1}}{\partial t}+{\boldsymbol{\nabla }}\cdot ({{\boldsymbol{u}}}_{{\boldsymbol{ins}}}{c}_{a1}) & = & {D}_{a1}{{\boldsymbol{\nabla }}}^{2}{c}_{a1}+\mathop{\underbrace{(\frac{{e}_{0}{K}_{a}-{({c}_{a1})}^{2}}{{K}_{a}}){R}_{a1}\,{L}_{EC}}}\limits_{production\,}\\  &  & -\mathop{\underbrace{{k}_{a1}^{+}\,{r}_{a}^{f}\,{c}_{a1}}}\limits_{bound\,to\,free\,Tie-2}+\mathop{\underbrace{{k}_{a1}^{-}\,{r}_{a1}^{a}}}\limits_{unbound\,from\,active\,Tie-2}-\mathop{\underbrace{{\varepsilon }_{a1}\,{c}_{a1}}}\limits_{excretion}\end{array}\end{array}$$

10 $$\begin{array}{ccc}\frac{{\rm{\partial }}{c}_{a2}}{{\rm{\partial }}t}+{\boldsymbol{\nabla }}.({{\boldsymbol{u}}}_{{\boldsymbol{i}}{\boldsymbol{n}}{\boldsymbol{s}}}{c}_{a2}) & = & {D}_{a2}{{\boldsymbol{\nabla }}}^{2}{c}_{a2}+\mathop{\underbrace{(\frac{{e}_{0}{K}_{a}-{({c}_{a2})}^{2}}{{K}_{a}}){R}_{a2}\,{L}_{EC}{L}_{TC}}}\limits_{\,production\,}\\  &  & -\mathop{\underbrace{{k}_{a2}^{+}\,{r}_{a}^{f}\,{c}_{a2}}}\limits_{bound\,to\,free\,Tie-2}+\mathop{\underbrace{{k}_{a2}^{-}\,{r}_{a2}^{a}}}\limits_{unbound\,from\,active\,Tie-2}-\mathop{\underbrace{{\varepsilon }_{a2}\,{c}_{a2}}}\limits_{excretion}\end{array}$$

11 $$\frac{d{r}_{a}^{f}}{dt}\,=-\,{k}_{a1}^{+}\,{r}_{a}^{f}\,{c}_{a1}+{k}_{a1}^{-}\,{r}_{a1}^{a}-{k}_{a2}^{+}\,{r}_{a}^{f}{c}_{a2}+{k}_{a2}^{-}\,{r}_{a2}^{a}$$

12 $$\frac{d{r}_{a1}^{a}}{dt}={k}_{a1}^{+}\,{r}_{a}^{f}\,{c}_{a1}-{k}_{a1}^{-}\,{r}_{a1}^{a}$$

13 $$\frac{d{r}_{a2}^{a}}{dt}={k}_{a2}^{+}\,{r}_{a}^{f}\,{c}_{a2}-{k}_{a2}^{-}\,{r}_{a2}^{a}$$

MMPs secreted by both ECs and TCs degrade ECM to make space available for TCs and ECs to spread^5,37^. Based on the proteolytic mechanism of MMPs, the coupled ADME equations of MMPs and ECM are presented in Eqs. 14 and 15, respectively,

14 $$\frac{\partial {c}_{m}}{\partial t}+{\boldsymbol{\nabla }}.({{\boldsymbol{u}}}_{{\boldsymbol{ins}}}{c}_{m})={D}_{m}\,{{\boldsymbol{\nabla }}}^{2}{c}_{m}+\mathop{\underbrace{{R}_{m,T}\,\vartheta \,{L}_{TC}}}\limits_{\begin{array}{c}production\,by\\ TCs\end{array}}+\mathop{\underbrace{{R}_{m,E}\,{L}_{EC}}}\limits_{\begin{array}{c}production\,by\\ ECs\end{array}}-\mathop{\underbrace{{\varepsilon }_{m}{c}_{m}}}\limits_{excretion}$$

15 $$\frac{\partial {c}_{e}}{\partial t}=-\mathop{\underbrace{{\varepsilon }_{e}\,{c}_{m}\,{c}_{e}}}\limits_{excretion}$$

${c}_{m}$ and ${c}_{e}$ are respectively concentrations of MMPs and ECM (fibronectin), ${R}_{m,T}$ and ${R}_{m,E}$ are respectively MMP secretion rates by TCs and ECs, ${\varepsilon }_{m}$ is natural excretion rate of MMPs and ${\varepsilon }_{e}$ is excretion rate of ECM by MMPs, respectively. We assume that MMP secretion by TCs (marked by *L*_*TC*_) dynamically varies based on cellular vitality,$\,\vartheta$.

### Cellular scale: agent-based cellular phenotypes

#### TC phenotypes

The concept of cellular vitality, $\vartheta$, is considered to determine TC activities and phenotypes. In the CR-based model presented in Eq. 16, the nutrients in the CR reaction, oxygen and glucose, increase $\vartheta$ and the waste product of the CR reaction, carbon-dioxide, decreases $\vartheta$. In this equation, *φ* is a proportionality coefficient, ${c}_{{o}_{2}}^{ch}$, ${c}_{g}^{ch}$, and ${c}_{co2}^{ch}$ are oxygen, glucose, and carbon dioxide characteristic concentrations, respectively^62^. $H({c}_{c{o}_{2}}-{c}_{c{o}_{2}}^{ch})\,$is a Heaviside function to ensure that carbon dioxide reduces $\vartheta$ when it’s concentration, ${c}_{c{o}_{2}}$, exceeds the characteristic value, ${c}_{c{o}_{2}}^{ch}$. In this model, the TCs with $\vartheta$ below$\,{\vartheta }^{ch}$are assumed to be quiescent (unable to migrate and proliferate) and those with $\vartheta$ above ${\vartheta }^{ch}$are active (prone to migrate and proliferate)^19^. As a biological assumption, the quiescent TCs can be converted to both proliferative and necrotic phenotypes based on their $\vartheta$ and cellular energy ($\psi$) values, respectively, and the proliferative TCs can become quiescent based on $\vartheta$; however, necrotic TCs cannot be converted to the other phenotypes. In the cellular energy model presented in Eq. 17, the quiescent TCs consume $\psi$ at a constant rate, ${k}_{q}^{c}$, but the active ones produce $\psi$ at a linear rate related to $\vartheta$ with a proportional coefficient, ${k}_{a}^{p}$; they also consume $\psi$ based on a M-M model with maximum rate ${k}_{a}^{c}$ and M-M’s constant 1^19^. Quiescent TCs with negative $\psi$ are converted to necrotic phenotype. The active TCs need a characteristic energy, ${\psi }^{ch}$, before they can proliferate into two daughter TCs with half value of ${\psi }^{ch}$^63,64^. Here, $\psi$ introduces available units of ATP molecules as a function of mitochondrial activity^19,61^. Based on the CR-reaction, a mathematical model of coupled cellular vitality and cellular energy is presented in Eqs. 16 and 17:

16 $$\vartheta =(\varphi \,\frac{{c}_{{o}_{2}}}{{c}_{{o}_{2}}+{c}_{{o}_{2}}^{ch}}\frac{{c}_{g}}{{c}_{g}+{c}_{g}^{ch}})\exp (-5{(\frac{{c}_{co2}}{{c}_{co2}^{ch}}-1)}^{4}H({c}_{c{o}_{2}}\,-\,{c}_{c{o}_{2}}^{ch}))$$

17 $$\frac{d\psi }{dt}=(\,{k}_{a}^{p}\vartheta -{k}_{a}^{c}\frac{\vartheta }{\vartheta +1}\,)H(\vartheta -{\vartheta }^{ch})-{k}_{q}^{c}H({\vartheta }^{ch}-\vartheta )$$

#### EC phenotypes

An agent-based model determines the proliferation, migration, quiescence persistence, and death of the tECs and sECs. The cell phenotypes affect neo-vessel growth dynamics during angiogenesis. The tECs migrate toward positive gradients of VEGF and ECM (fibronectin)^34,36,65^. The sECs migrate into the tECs-generated conduits in ECM and also proliferate and create lumens of the neo-vessels^5,33,65^. Moreover, the sECs can differentiate into tECs in response to high VEGF concentration, and thus generate bifurcating branches from the neo-vessel wall^19,65^. The inactive quiescence and death states are also considered for sECs based on VEGF and Ang-1/Ang-2 concentrations (see Eq. 21).

### Tissue scale: solid tumor growth and angiogenesis

Fibronectin is a provisional ECM component abundant in tumors that serves as an adhesive glycoprotein and modulates mechanical stiffness. It is important for cell adhesion and migration and supports haptotactic migration of adhesive cells, including tECs and active TCs^36^. tECs can also use chemotaxis to migrate in response to VEGF gradients^34,65,66^. Moreover, any live cell can randomly walk in the ECM due to intracellular actomyosin-cytosolic dynamics and extracellular stimuli^67–70^. Corresponding to these movement mechanisms of tECs and TCs, the tumor growth and angiogenesis are mathematically modeled by Eqs. 18 and 19, respectively. Implementation of the DHCD method to calculate the highest DOF movement probabilities for each cell (tumor and endothelial cells) derived from these equations is presented in Supplementary Material^71^.

18 $$\frac{\partial {\rho }_{EC}}{\partial t}=\mathop{\underbrace{\,{D}_{EC}\,{{\boldsymbol{\nabla }}}^{2}{\rho }_{EC}}}\limits_{Random\,walk}-{\boldsymbol{\nabla }}.(\mathop{\underbrace{\frac{{\beta }_{c}}{1+\alpha {c}_{v}}{\rho }_{EC}{\boldsymbol{\nabla }}{c}_{v}}}\limits_{chemotaxis}+\mathop{\underbrace{{\beta }_{h}\,{\rho }_{EC}{\boldsymbol{\nabla }}{c}_{e}}}\limits_{Haptotaxis})$$

19 $$\frac{\partial {\rho }_{TC}}{\partial t}=\mathop{\underbrace{{D}_{TC}{{\boldsymbol{\nabla }}}^{2}{\rho }_{TC}}}\limits_{Random\,walk}-{\boldsymbol{\nabla }}.(\mathop{\underbrace{{\beta }_{h}\,{\rho }_{TC}{\boldsymbol{\nabla }}{c}_{e}}}\limits_{Haptotaxis})$$

${\rho }_{EC}$ and ${\rho }_{TC}$ are respectively EC and TC densities, ${D}_{EC}$ and ${D}_{TC}$ are respectively diffusivity of ECs and TCs in the interstitium, $\alpha$ is the saturation coefficient for chemotaxis, and ${\beta }_{c}$ and ${\beta }_{h}$ are weight coefficients for chemotaxis and haptotaxis, respectively.

Accumulation of rapidly dividing TCs increases the mechanical, compressive solid stress, ${p}_{s}$^72–74^ For this additional tumor growth-induced stress, we implement a Gaussian-like function for accumulative systems^19^. Tumor growth-induced solid stresses can also influence tumor growth and angiogenesis. Therefore, we assume that TCs are pushed toward low pressure/stress regions and tumor-induced solid stress acts as a barrier for tEC migration^73,75,76^.

The direct effect of IFF is to drive mass transport of molecular agents. It is generally thought that the pore size of the interstitium is too small for large object such as cells to be carried with the IFF. However, because cell migration is influenced by molecular gradients, IFF indirectly affects cell migration by affecting chemotaxis. Although we and others have shown that IFF can be a signal for cell migration or differentiation^7,77,78^, this mechanism was not a focus of this study. Therefore, we assume that the interstitial flow doesn’t directly affect the cellular dynamics and phenotype.

### Tissue scale: vessel growth and remodeling

After new vessels form via angiogenesis, they need to form lumens before flow can proceed. They do this through a process of lumenogenesis, which is controlled by VEGF, Ang-1, and Ang-2. The equation for neo-vessel diameter, *d*_*v*_, is written as Eq. 20,

20 $${d}_{v}=\frac{{G}_{s}({c}_{v},{c}_{a1},{c}_{a2})}{{G}_{s}({c}_{v},{c}_{a1},{c}_{a2})+{G}_{0}}{d}_{c}$$

${G}_{s}$ is the stalk endothelial growth function controlling lumenogenesis, and ${G}_{0}$ is a M-M constant for neo-vessel lumen growth. ${G}_{s}$ depends on the proliferation, maturation, and death rate of sECs according to^79^:

21 $$\begin{array}{ccc}\frac{d{G}_{s}}{dt} & = & \mathop{\underbrace{({\alpha }_{p}\,\frac{{c}_{v}}{{c}_{v}+{\theta }_{p}})}}\limits_{sEC\,proliferation}-\mathop{\underbrace{({\omega }_{m}\frac{{c}_{v}}{{c}_{v}+{\theta }_{m}})\,(\frac{(\frac{{c}_{a1}}{{c}_{a2}})}{(\frac{{c}_{a1}}{{c}_{a2}})+{\rho }_{m}})}}\limits_{sEC\,quiescence-for-maturation\,}\\  &  & \,-\mathop{\underbrace{(1-\frac{{c}_{v}}{{c}_{v}+{\theta }_{d}})\delta \,}}\limits_{sEC\,death}+{k}_{age}\end{array}$$

${\alpha }_{p}$ and ${\theta }_{p}$ are, respectively, the maximum rate and the M-M constant for sEC proliferation; ${\omega }_{m}$, ${\theta }_{m}$, and ${\rho }_{m}$ are the maximum quiescence persistence rate - which is a parameter that controls how long the cell remains quiescent before maturing to form lumen (quiescence-for-maturation) - for sEC, the M-M constants for maturation relative to VEGF and Ang-1/Ang-2, respectively; $\delta$ and ${\theta }_{d}$ are the maximum rate and M-M constant for sEC death, respectively. ${k}_{age}$ is also a positive constant that represents the neo-vessel lumen growth due to aging^19^.

#### Vessel adaptation

Angiogenic neo-vessels are highly sensitive to biomechanical and biochemical stimuli in the TME. Respectively shown in Eq. 22a–d, the TME stimuli caused by endothelial wall shear stress (WSS), *S*_*WSS*_, transvascular pressure, *S*_*p*_, hematocrit-induced metabolite, *S*_*HM*_, and VEGF, *S*_*vegf*_, are the major signals that control neo-vessel dilation/constriction^21,32,80–82^.

22a $${S}_{WSS}={\log }_{10}(\tau +{\tau }_{ref})$$

22b $${S}_{p}=-\,{k}_{p}{\log }_{10}(100-86.\exp \,(\,-\,5000\,{(lo{g}_{10}(lo{g}_{10}(p)))}^{5.4}))$$

22c $${S}_{HM}={k}_{m}{\log }_{10}(\frac{{Q}_{ref}}{{Q}_{lum}\,{H}_{D}}+1)$$

22d $${S}_{vegf}=-\,{k}_{e}{\log }_{10}(\frac{({k}_{0}\,\frac{{c}_{v}^{ch}}{{c}_{v}^{ch}+{c}_{v}})({d}_{v}-{d}_{0})}{{\tau }_{c}}+1)$$

In Eq. 22a, $\tau$ is the WSS in a neo-vessel segment, which depends on blood dynamic viscosity, ${\mu }_{blood}$, lumen flow rate, ${Q}_{lum}$, and vessel diameter, ${d}_{v}$. It is calculated using Hagen-Poiseuille’s law: $\tau =\frac{32\,{\mu }_{blood}\,{Q}_{lum}}{\pi \,{d}_{v}^{3}}$. ${\tau }_{ref}$ is a positive constant to avoid singularity at low WSS. In Eq. 22b, ${k}_{p}$ is a proportional coefficient and *p* is transvascular pressure. In Eq. 22c, ${k}_{m}$ is a proportional constant describing the intensity of metabolic stimuli, ${Q}_{ref}$ is the maximum flow rate within the neo-vessel network, determined by the inflow supplied by the primary vessels^83^; ${H}_{D}$ is the hematocrit of blood, and ${Q}_{lum}$ is the blood flow rate within the lumen. In Eq. 22d, ${k}_{e}$ is a proportionality coefficient, ${k}_{0}$ the vessel wall elasticity constant per unit length in the absence of VEGF, ${c}_{v}^{ch}\,$ is the characteristic VEGF concentration for which the vessel wall susceptibility to adaptation defined by ${\tau }_{e}({d}_{v},\,{c}_{v})\,={k}_{0}\frac{{c}_{v}^{ch}}{{c}_{v}^{ch}+{c}_{v}}({d}_{v}-{d}_{0})$ is half the maximum value in the absence of VEGF, (${\tau }_{e}({d}_{v},{c}_{v}^{ch})\,=\frac{1}{2}{\tau }_{e}^{max}({d}_{v},0)\,=\frac{1}{2}{k}_{0}({d}_{v}-{d}_{0})$), ${d}_{0}$ stress-free vessel diameter, and ${\tau }_{c}$ is a characteristic elastic stress introduced to ensure calculation stability^21^.

Finally, the TME-induced adaptation of vessel diameter is considered in Eq. 23. In addition to four TME stimuli, a shrinkage term, ${S}_{Sh}$, is included to allow the possibility for vessels and decrease their diameter if the other stimuli are not sufficient for growth^21,82^.

23 $$\frac{d({d}_{v})}{dt}=({S}_{WSS}+{S}_{p}+{S}_{HM}+{S}_{vegf}-{S}_{Sh})$$

#### Vessel deformation

We assume that the neo-vessels are mechanically compliant with a constant elasticity, $E$ and compliance power, $cp$, as well as a collapse pressure, ${p}_{c}$^55,84,85^. Therefore, the transvascular pressure, $p$, and tumor growth-induced solid stresses, ${p}_{s}$, through Eq. 24 can deform the neo-vessels and change the vessel diameter from ${d}_{v}$, to a deformed vessel diameter, ${d}_{v}^{d}$,

24 $${d}_{v}^{d}={d}_{v}{(\frac{p-{p}_{s}+{p}_{c}}{E})}^{cp}$$

#### Vessel disruption

In addition to vessel growth and remodeling mechanisms, we consider the possibility of vessel disruption. If a neo-vessel has insufficient blood flow and τ falls below a threshold value, ${\tau }_{th}$, during a survival time, ${t}_{s}$, then the vessel is mathematically eliminated^20,42,86^. Similarly, vessels inside the tumor exposed to VEGF concentrations higher than a threshold value, ${c}_{v}^{th}$, during the survival time, ${t}_{s}$, are also disrupted^21^.

### Tissue scale: Hemodynamics and interstitial fluid flow in the TME

For the fluid dynamics model, we consider flow in three regions spanning from a neo-vessel’s lumen to the interstitium: intravascular blood flow, transvascular fluid flow, and interstitial fluid flow (IFF). Because of the very small diameter of newly-generated vessels, the Womersley number of flow is very small, and hence the pulsatile effects of the cardiac cycle can be ignored^87,88^. Therefore, Hagen-Poiseuille’s law as an exact solution of the Navier-Stokes fluid dynamics equation can be applied for intravascular blood flow. IFF is calculated using Darcy’s law assuming low porosity of healthy and tumor tissues^18,22,52,87^. Finally, transvascular fluid flow is calculated by Starling’s law, which couples IFF and intravascular blood flow^18,22,52^. The continuity equation for intravascular blood flow is written as Eq. 25. In this equation, Q_lum_ is the blood flow rate in the lumen calculated as the difference between intravascular blood flow rate based on Hagen-Poiseuille’s law, Q_IBF_, and transvascular fluid flow rate based on Starling’s law, Q_TFF_, respectively, shown in Eqs. 26 and 27.

25 $${\sum }_{b=1}^{N}{Q}_{lum}^{b}{\beta }^{b}=0,\,{Q}_{lum}={Q}_{IBF}-{Q}_{TFF}$$

26 $${Q}_{IBF}=\frac{\pi }{128}\frac{\varDelta {p}_{lum}{{d}_{v}}^{4}}{L\,{\mu }_{blood}({d}_{v},{H}_{D})}$$

27 $${Q}_{TFF}=\pi {d}_{v}\,L\,{L}_{p}(p-({\pi }_{lum}-{\pi }_{ins})\sigma )$$

In Eq. 25, N = 26 is the number of peripheral vessel lattice nodes adjacent to the central vessel node, and $\beta$ describes the direction of lumen blood flow; it is +1 for outlet flow from a peripheral node and −1 for inlet flow to a peripheral node. In Eq. 26, ${p}_{lum}$ is intravascular blood pressure, ${d}_{v}$ is the neo-vessel diameter, *L* is the length of a neo-vessel segment which can be equal to 1, $\sqrt{2}$, or $\sqrt{3}$ times the lattice length up to the neo-vessel path line in three-dimensional space, and ${\mu }_{blood}$ is the dynamic viscosity of non-Newtonian blood as a function of neo-vessel diameter, ${d}_{v}$, and blood hematocrit, ${H}_{D}$. In Eq. 27, ${L}_{p}$ is the hydraulic conductivity of the neo-vessel wall, which is a constant for healthy tissue and increases in tumor tissue based on vessel diameter^20,55^; $p$ is transvascular pressure, $\sigma$ is the osmotic reflection coefficient, ${\pi }_{lum}$ and ${\pi }_{ins}$ are colloid osmotic (oncotic) pressures of the intravascular plasma and interstitial fluid, respectively. Oncotic pressure is a type of osmotic pressure that depends on a concentration difference across a semipermeable membrane structure. Oncotic pressure can only be transmitted by the fluid phase of the tissue, and not through solid elements such as matrix, cells, or the vessel wall^89^.

The continuity equation for IFF given in Eq. 28 shows both incompressibility of plasma fluid by zero divergence of interstitial fluid velocity, ${u}_{ins}$, and leakiness of the neo-vessel wall due to a source term in vascular tissue. Darcy’s law determining IFF is also shown in Eq. 29.

28 $${\boldsymbol{\nabla }}.{{\boldsymbol{u}}}_{{\boldsymbol{ins}}}=\{\begin{array}{ll}{L}_{p}\,S/V\,(p-({\pi }_{lum}-{\pi }_{ins})\sigma ) & At\,the\,vessel\,wall\\ 0\, & In\,the\,extravascular\,space\end{array}$$

29 $${{\boldsymbol{u}}}_{{\boldsymbol{ins}}}=-\,{K}_{ins}{\boldsymbol{\nabla }}{p}_{ins}$$

${u}_{ins}$ is the velocity of IFF, ${K}_{ins}$ is the interstitial hydraulic conductivity (IHC) of the TME, $S/V$ is the surface area of the neo-vessel per unit volume for mass transport in the interstitium^18,50^. By replacing Eq. 28 in Eq. 29, we can derive the Poisson-Laplace’s equation for IFP shown in Eq. 30.

30 $$-{{\boldsymbol{\nabla }}}^{2}{p}_{ins}=\{\begin{array}{ll}{L}_{p}\,\frac{S/V}{{K}_{ins}}((p-({\pi }_{lum}-{\pi }_{ins})\sigma )) & At\,the\,vessel\,wall\\ 0 & In\,the\,extravascular\,space\end{array}$$

### Tissue scale: hemorheology

Because of the non-Newtonian behavior of blood, its dynamic viscosity as a function of vessel diameter, d_v_, and blood hematocrit, H_D_, is calculated using Eq. 31. Indeed, we assume that blood is a non-Newtonian suspension red blood cells within a Newtonian plasma fluid^57,82,83,90^. In Eq. 31, ${\mu }_{blood}$ and ${\mu }_{plasma}\,$ are the dynamic viscosity of blood and plasma, respectively; ${\mu }_{n}$ is the relative dynamic viscosity of healthy blood with a normal hematocrit, ${H}_{D}^{n}$, defined in Eq. 32 as a function of vessel diameter, ${d}_{v}$, and $f({H}_{D})$ is a function relating viscosity to hematocrit, see Eq. 33.

31 $${\mu }_{blood}({d}_{v},{H}_{D})=(1+({\mu }_{n}-1)f({H}_{D}){(\frac{{d}_{v}}{{d}_{v}-1.1})}^{2}){(\frac{{d}_{v}}{{d}_{v}-1.1})}^{2}{\mu }_{plasma}$$

Equation 31 is an empirical formula first presented by Pries, *et al*.^80^ based on the behavior of blood in very small vessels.

32 $${\mu }_{n}=3.2+6\,\exp \,(\,-\,0.085{d}_{v})\,-2.44\,\exp (\,-0.06\,{{d}_{v}}^{0.645})$$

33 $$\begin{array}{c}f({H}_{D})=\frac{{(1-{H}_{D})}^{C}-1}{{(1-{H}_{D}^{n})}^{C}-1}\\ C=(0.8+\exp (\,-\,0.075\,{d}_{v}))(\frac{1}{1+{10}^{-11}{{d}_{v}}^{12}}-1)+\frac{1}{1+{10}^{-11}{{d}_{v}}^{12}}\end{array}$$

The hematocrit distribution can change at bifurcations. Generally, the faster daughter branch (branch 1) has more hematocrit^18,90^. If the velocity ratio of two branches exceeds a threshold value, ${r}_{th}$, all the blood cells enter the faster daughter branch^18,90^. According to these assumptions, the relation between the hematocrit of the parent vessel, ${{H}_{D}}^{p}$, and the daughter branches, ${{H}_{D}}^{1}$ and ${{H}_{D}}^{2}$, are respectively written as Eqs. 34–36 based on the velocity ratio of two branches, ${u}_{lum}^{1}/{u}_{lum}^{2}$,

34 $${{H}_{D}}^{p}={{H}_{D}}^{1}+{{H}_{D}}^{2}$$

35 $$if\,\frac{{u}_{lum}^{1}}{{u}_{lum}^{2}} < {r}_{th}\,\to \{\begin{array}{l}{{H}_{D}}^{1}={(1+\frac{{u}_{lum}^{1}}{2{u}_{lum}^{2}})}^{-1}\,{{H}_{D}}^{p}\\ {{H}_{D}}^{2}=\,{(1+\frac{2{u}_{lum}^{2}}{{u}_{lum}^{1}})}^{-1}\,{{H}_{D}}^{p}\end{array}$$

36 $$if\frac{{u}_{lum}^{1}}{{u}_{lum}^{2}} > {r}_{th}\to \{\begin{array}{l}{{H}_{D}}^{1}={{H}_{D}}^{p}\\ {{H}_{D}}^{2}=0\end{array}$$

## Computational Implementation

### Computational geometry

**Figure 2:**
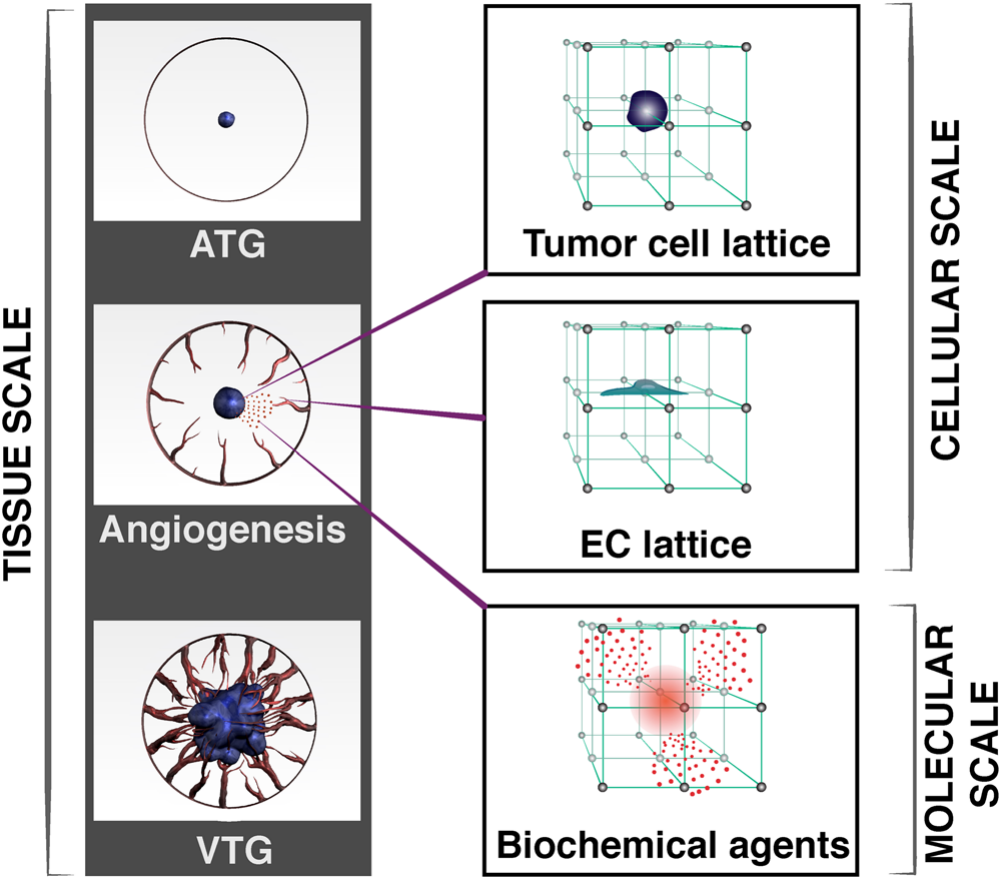
Schematic of the three-dimensional multi-scale TME. The model uses different computational approaches for the various species at the subcellular, cellular and tissue size scales. Subcellular scale; finite difference mesh for each biochemical agent. Cellular scale; two lattices for TCs and ECs with the same grid as finite difference mesh. Tissue scale; the host vasculature is represented as a circle surrounding the tumor. Sprouts initiate stochastically from this location. A pressure boundary condition is imposed at this circle, providing a source for blood flow into the angiogenic vessel network. Tumor tissue starts growing at the center of the computational domain.

We selected a 10 mm cube with 201 × 201 × 201 lattice nodes for the computational domain of the three-dimensional TME. A three-dimensional double hybrid continuous-discrete (DHCD) method was applied to solve the mathematical equations of the TME model (see Supplementary Material). In Fig. 2, a schematic of the multi-scale TME including subcellular, cellular and tissue scales is shown. Two separate cellular lattices with 3 × 3 × 3 nodes are constructed to determine the location of each tumor and endothelial cell, and a finite difference mesh with the same size as the lattices is selected to solve continuous parts of the DHCD (see Supplementary Material). The distance between adjacent nodes is 50 µm, which means we can assume each cell is like a sphere with diameter 50 µm or a cube with side 50 µm. The equations for the continuous part of the DHCD (all model equations except Eqs. (18) and (19) for tEC and TC, respectively) are normalized, discretized, and solved by a finite difference method (FDM) to determine the spatiotemporal distributions of TME biochemical and biomechanical factors. Based on these distributions, the motion probabilities of each TC and tEC are calculated to determine tumor progression in the TME (see Supplementary Material online for detailed computational approach and model setup). The parameters of the model used for computational results have been listed in Table S of Supplementary Material. The mathematical model is completely robust and independent of the selected parameters and thus can be used to simulate various tumor types with specific conditions. We demonstrate the robustness of model by changing some parameters, which can represent some of the most likely clinical problems of cancer patients.

For the lattice resolution, we checked the mesh independency by performing simulations on lattices 20% smaller and 20% larger than the 50 µm used in the present study. Reducing the lattice size by 20% changed results by less than 10%; therefore, to reduce computational cost we chose the 50 µm lattice to present the results. A 50 µm spacing is also consistent with the average size of cancer and endothelial cell.

### Initial and boundary conditions

The initial concentrations of glucose, oxygen, carbon-dioxide, and ECM were assumed to be homogeneous and equal to ${c}_{g}^{ch}$, ${c}_{{o}_{2}}^{ch}$, ${c}_{c{o}_{2}}^{ch}$, and ${c}_{e}^{ch}$, respectively. The initial concentrations of VEGF, Ang-1, Ang-2, and MMP are assumed to be zero. The free and active VEGFR-2 and Tie-2 are also homogeneously initialized to zero. At the boundaries of the computational domain of the TME, a Dirichlet boundary condition was used for each agent with value equal to its initial concentration. The TME was seeded with five tumor cells located at the center of the computational cube and a hypothetical circular primary vascular network with a radius approximately 5 mm. The locations of initial sprouts on the circle of primary vessels are determined based on VEGF concentration but spaced randomly according to NOTCH induction more than 50 µm apart^21^. The biomechanical factors IFF velocity and IFP, intravascular blood flow velocity and pressure, and WSS were initially set zero in the entire computational domain and boundaries. For these parameters, a Dirichlet boundary condition with zero value was set on all boundaries of the TME domain. At the inlet of the neo-vessels connected to the primary vessels, we chose *p*_*lum*_ = 30 mmHg, consistent with the range of primary vessel pressures reported in the literature^19,21,50,56^. To cover all three biological stages of tumor progression including ATG, angiogenesis (ATG-to-VTG), and VTG, we simulated 35 days of tumor growth and angiogenesis.

## Results and Discussion

### Morphology of the TME

**Figure 3:**
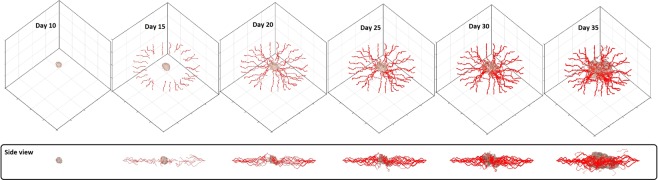
Variable morphologies of a three dimensional solid tumor and angiogenic neo-vessel network at days 10, 15, 20, 25, 30, and 35, reproducing avascular growth, angiogenesis, and vascular growth.

**Figure 4:**
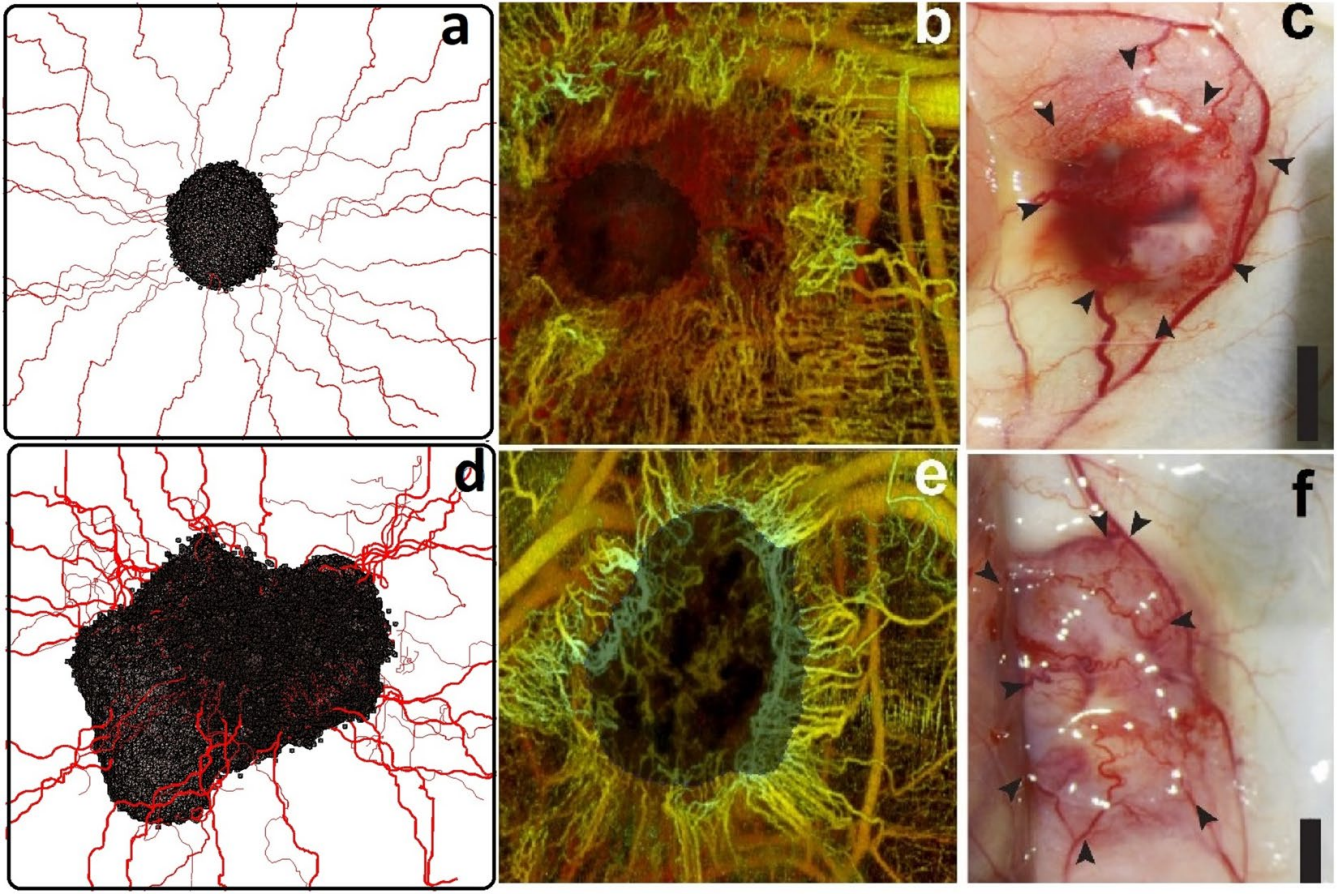
Biological validation of the systems biology model. Our simulations predict that tumor morphology remains symmetric through early ATG (**a**), and this resembles *in vivo* observations by Vakoc, *et al*.^92^ (**b**) and Roudnicky, *et al*.^91^ (**c**) Random penetration of vessels in VTG and the resulting nutrient supply causes asymmetry in tumor growth, predicted by the model (**d**) and also observed by Vakoc, *et al*.^92^ (**e**) and Roudnicky, *et al*.^91^ (**f**) Reprinted by permission from [Springer Nature Customer Service Centre GmbH]: [Springer Nature] [Oncogene] [Alternative transcription of a shorter, non-anti-angiogenic thrombospondin-2 variant in cancer-associated blood vessels, Filip Roudnicky, Sun Young Yoon, Susanna Poghosyan, Simon Schwager, Cedric Poyet, Giorgia Vella, Samia B Bachmann, Sinem Karaman, Jay W Shin, Vivianne I Otto], [2018].

To analyze the various stages of tumor growth and angiogenesis, we selected days 10, 15, 20, 25, 30, and 35, which span ATG, angiogenesis (ATG-to-VTG), and VTG (Fig. 3) (see Movie S1 for a video of three-dimensional tumor growth and angiogenesis). Before day 10, tumor cells are supplied by nutrient diffusion symmetrically, and they expand as a symmetric semi-spherical structure. Between days 10–15, VEGF produced by the tumor creates a gradient that reaches the primary vessel and causes sprouting angiogenesis to generate neo-vessels. The neo-vessels elongate, branch, deform and extend toward the tumor during days 15–25. After day 25, the ATG-to-VTG transition is completed as neo-vessels penetrate into the tumor. During VTG (days 25–35), heterogeneities emerge because of the non-uniform neo-vasculature and the resulting asymmetric distributions of nutrients and growth factors. This causes spatial variations in cancer cell proliferation, resulting in distortion of the semi-spherical geometry. This transition from symmetric to asymmetric tumor shape mimics the evolution of an actual tumor. A qualitative comparison between our results and two biological observations of solid-tumor morphology^91,92^ is illustrated in Fig. 4. Panels a–c show the initial stage of growth, with ATG transitioning to VTG, with very low density of angiogenic neo-vessels. Subsequently, VTG is characterized by a dense network of tumor-penetrating neo-vessels (Fig. 4d–f). In agreement with *in vivo* observations, our simulations demonstrate that VTG can be characterized by an asymmetric and more invasive morphology compared to the symmetric, spheroidal morphology of ATG. In addition to recapitulation of three major stages of tumor progression (ATG, angiogenesis and VTG), deconstructing a reliable morphology of a solid tumor is the most important goal of a three-dimensional computational model because asymmetries and heterogeneities create complex chemical and mechanical gradients that greatly influence tumor biology and physiology.

**Figure 5:**
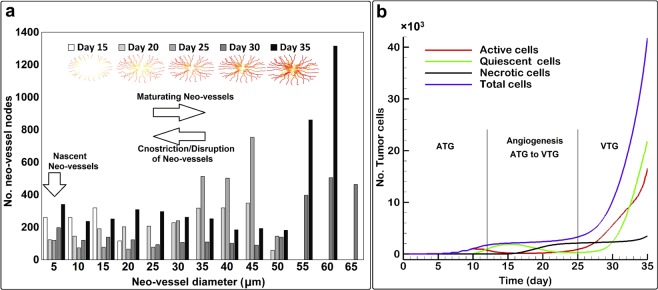
Dynamics of vessel diameter distribution (**a**) and cancer cell populations (**b**) during the three stages of tumor growth and angiogenesis (ATG, angiogenesis and VTG).

We next quantified the number of angiogenic neo-vessels and their diameters at different times during tumor growth (Fig. 5a). Nascent neo-vessels have diameters less than 5 µm, while subsequent growth and maturation increase vessel diameters and cause lumenogenesis, all regulated by environmental stimuli. Vessel diameters can also decrease if there is a lack of positive angiogenic stimuli (see Eqs. 22a–d and 23). Deformation of the compliant neo-vessel can be caused by tumor growth-induced solid stresses, which are generally opposed by transvascular fluid pressure gradients (see Eq. 24). Vessel integrity can also be compromised by low levels of WSS or high levels of VEGF, resulting in local changes in vessel diameter. During ATG, some neo-vessels grow to as large as 50 µm in diameter with the majority falling in the diameter range of 40–45 µm (Fig. 5a). During VTG, branching of the neo-vessels increases near the tumor boundary and inside the tumor where VEGF concentration is high (Fig. 5a). This is because the VEGF induces sECs of neo-vessels to differentiate into tECs and generate new branches. Behind the leading edge of angiogenic sprouts, vessel maturation causes an overall increase in vessel diameters, with some reaching 65 µm in diameter. In contrast, further flow-mediated vessel remodeling from Day 30 to Day 35 causes a reduction in the largest diameters from 65 µm to 60 µm (see Eq. 23).

In addition to vessel morphology, we can examine the evolution of cancer cell phenotypes in the simulations (Fig. 5b). During ATG, active cells proliferate, but nutrient limitations in the tumor center cause some cells to become quiescent starting around Day 10. As angiogenic neo-vessels approach the tumor, but before tumor vascularization, the active cells have completely converted to quiescent cells because of insufficient nutrients, and the total number of cells plateaus; later in the transition from ATG to VTG, some quiescent cells become necrotic due to chronic nutrient deprivation (see Eqs. 16 and 17, and section 2.2). The number of necrotic cells increases until nutrient concentrations start to rise again due to tumor vascularization. Interestingly, the necrotic core persists due to rapid growth at the tumor periphery, which depletes nutrients before they can reach the deeper cells. During VTG, vessels bring more nutrients, and active cells begin to proliferate. After a time lag of a few days, the quiescent cell population increases in a similar fashion, and finally the necrotic cell population starts to increase again. The total number of cells linearly increases during ATG, plateaus during angiogenesis (ATG-to-VTG), and then exponentially increases during VTG. All these four tumor population dynamics agree with experimental observations and computational analyses^19,30,55,93^ and are typical of solid tumors initiated far from primary-vessels^43,56^.

### Distribution of angiogenesis-associated TC vitality

**Figure 6:**
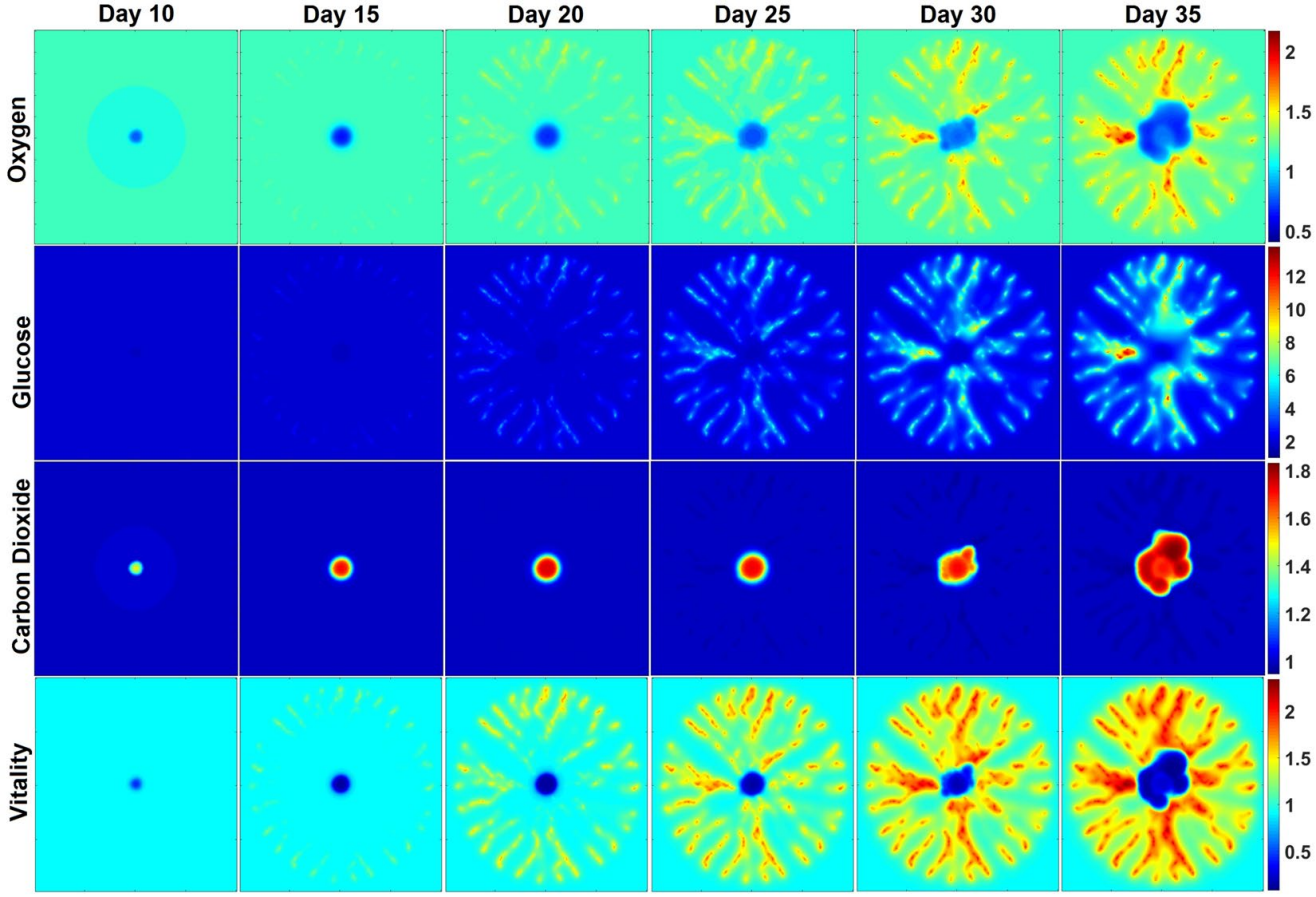
Spatial distributions of oxygen, glucose, carbon dioxide, and cellular vitality at the middle cross-section of the three-dimensional domain at days 10, 15, 20, 25, 35 of tumor progression.

We next examined the distributions of oxygen, glucose, CO_2_, and cellular vitality during tumor growth and angiogenesis (Fig. 6). The transition of the tumor geometry from a symmetric, semi-spheroid to an asymmetric, amorphous mass is apparent in the results. The active and quiescent TCs located at the tumor periphery regions around the necrotic core – central spherical region consistent during days 25–35 - (see Fig. 6, “Vitality”), consume more oxygen and glucose and, consequently, secrete more CO_2_. The limited supply of oxygen combined with high oxygen consumption by the peripheral TCs results in insufficient oxygen inside the tumor. However, glucose can be carried by neo-vessels to the boundary, but not to the central regions. Glucose delivered by the neo-vessels is sufficient for the peripheral cells, but not the central core of the tumor (Fig. 6, “Glucose”). The production rate of CO_2_ by the peripheral TCs is higher than its removal by intratumoral neo-vessels, so CO_2_ is concentrated inside the tumor, as shown in Fig. 6, “Carbon Dioxide”. Diffusion into the center with no vessels for convection/removal combined with inactivity of necrotic cells for production/consumption causes some accumulation of CO_2_, oxygen and glucose in the tumor necrotic core. Distributions of other biochemical agents and biomechanical factors within the TME are presented in Supplementary Material.

### Effect of biochemical blood abnormalities on tumor progression

**Figure 7:**
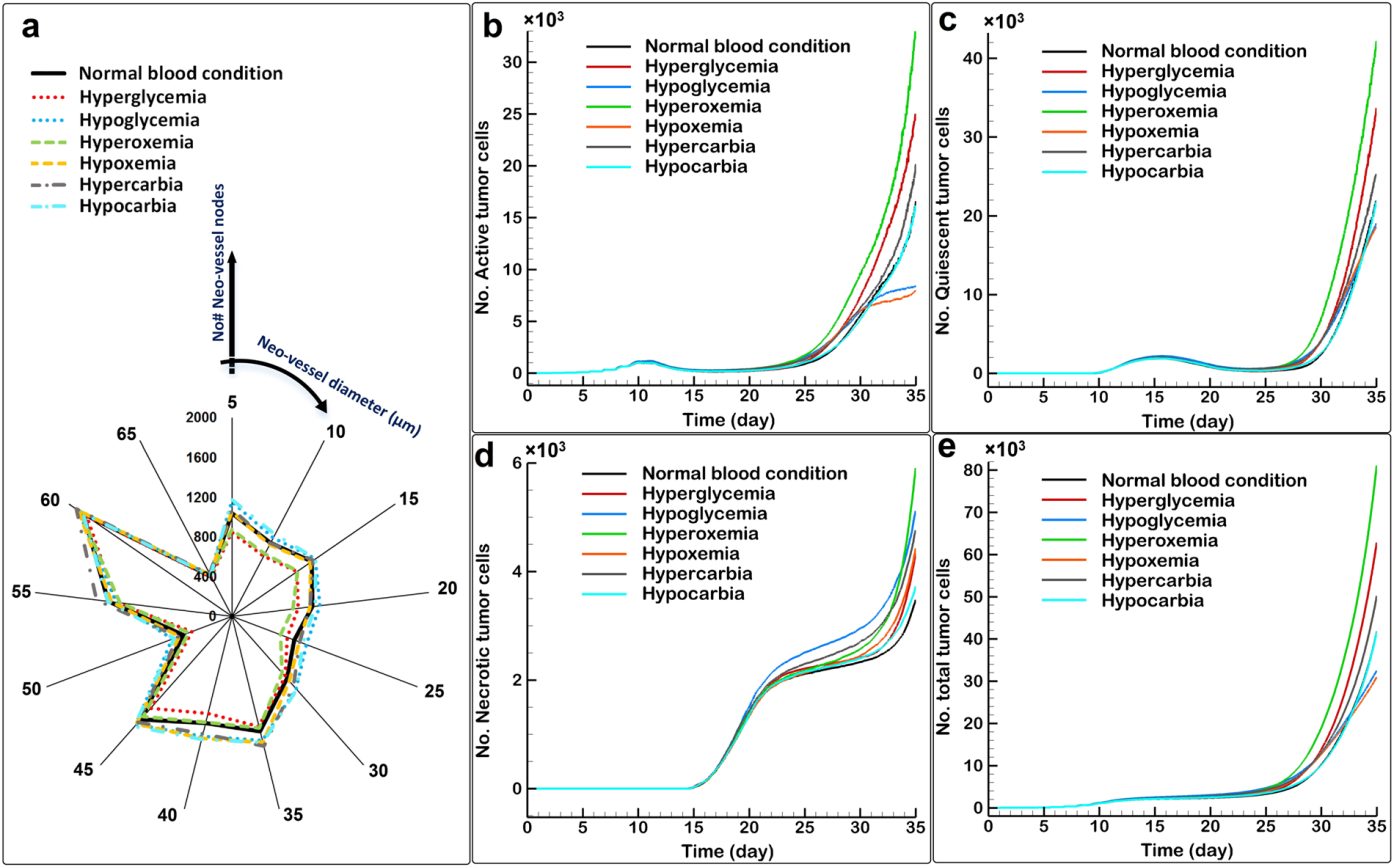
Effects of biochemical blood abnormalities on tumor progression, (**a**) distribution of angiogenic neo-vessels, (**b**) active tumor cells, (**c**) quiescent tumor cells, (**d**) necrotic tumor cells, (**e**) total tumor cells.

Because cancer patients often have abnormalities in their blood chemistry due to tumor growth or treatments, we next used the model to predict how these abnormalities might affect evolution of the tumor and its vasculature. We simulated hyperglycemia by doubling the glucose perfusion rate and hypoglycemia by halving the glucose perfusion rate. Hyperoxemia and hypoxemia were simulated by doubling and halving the oxygen perfusion rate, respectively, and hypercarbia and hypocarbia were produced by halving and doubling the carbon-dioxide perfusion rate, respectively. These conditions were compared with each other, and with normal blood (Fig. 7). Hyperoxemia and Hyperglycemia resulted in similar neo-vessel distributions, with the fewest vessels of all the conditions (Fig. 7a). This is because tissues with high levels of oxygen and glucose do not need to recruit as many new vessels to supply the tumor. With hypoxemia and hypercarbia vessel densities are similar, but hypercarbia resulted in more neo-vessel maturation. Hypoglycemia and hypocarbia both resulted in maximum neo-vessel number, with similar distributions. The conditions that produced angiogenesis that most resembled normal blood in terms of sprouting and branching were hypoxemia and hypercarbia. Considering neo-vessel maturation, hyperoxemia produced results similar to normal blood. Examining the populations of vessels that completely matured, all cases except hypercarbia have approximately equal vessel distribution. It is noteworthy that these differences in angiogenic neo-vessel distributions emerge because each blood abnormality affects a specific aspect of cellular respiration, which affects oxygen availability to the TCs and, consequently, tumor-induced VEGF.

Before VTG commences, blood abnormalities have no significant effect on the numbers of active, quiescent, necrotic and, consequently, total tumor cell (Fig. 7b–e). However, at the end of the transition from ATG to VTG, small effects of the abnormalities – especially on necrotic cells in the hypoglycemia blood condition – can be seen. Hypoglycemia and hypoxemia reduce the active and quiescent cells in the same manner, but hypoglycemia increases necrotic cells more than hypoxemia. These results suggest that hypoglycemia has a more potent positive effect on tumor growth than hypoxemia. Overall, these abnormalities reduce the total tumor cells (Fig. 7e). Hyperoxemia, and to a lesser extent hyperglycemia, increase active, quiescent, necrotic, and thus total cells. Interestingly, the necrotic core increases in size in response to both low and high oxygen/glucose conditions. In the case of low oxygen or glucose, this is probably due to insufficient penetration of nutrient-carrying vessels into the core. In the case of high oxygen or glucose, it is because of nutrient depletion by the outer layers of tumor cells, which starves the deeper cells. As another factor for promoting tumor growth, hypercarbia slightly increases active and quiescent cells and more strongly increases necrosis due to accumulation of more carbon-dioxide inside the tumor, leading to cell toxicity and death. In our simulations, hypocarbia produces tumor growth similar to the normal condition.

### Effect of blood pressure and interstitial hydraulic conductivity (IHC) on tumor progression

**Figure 8:**
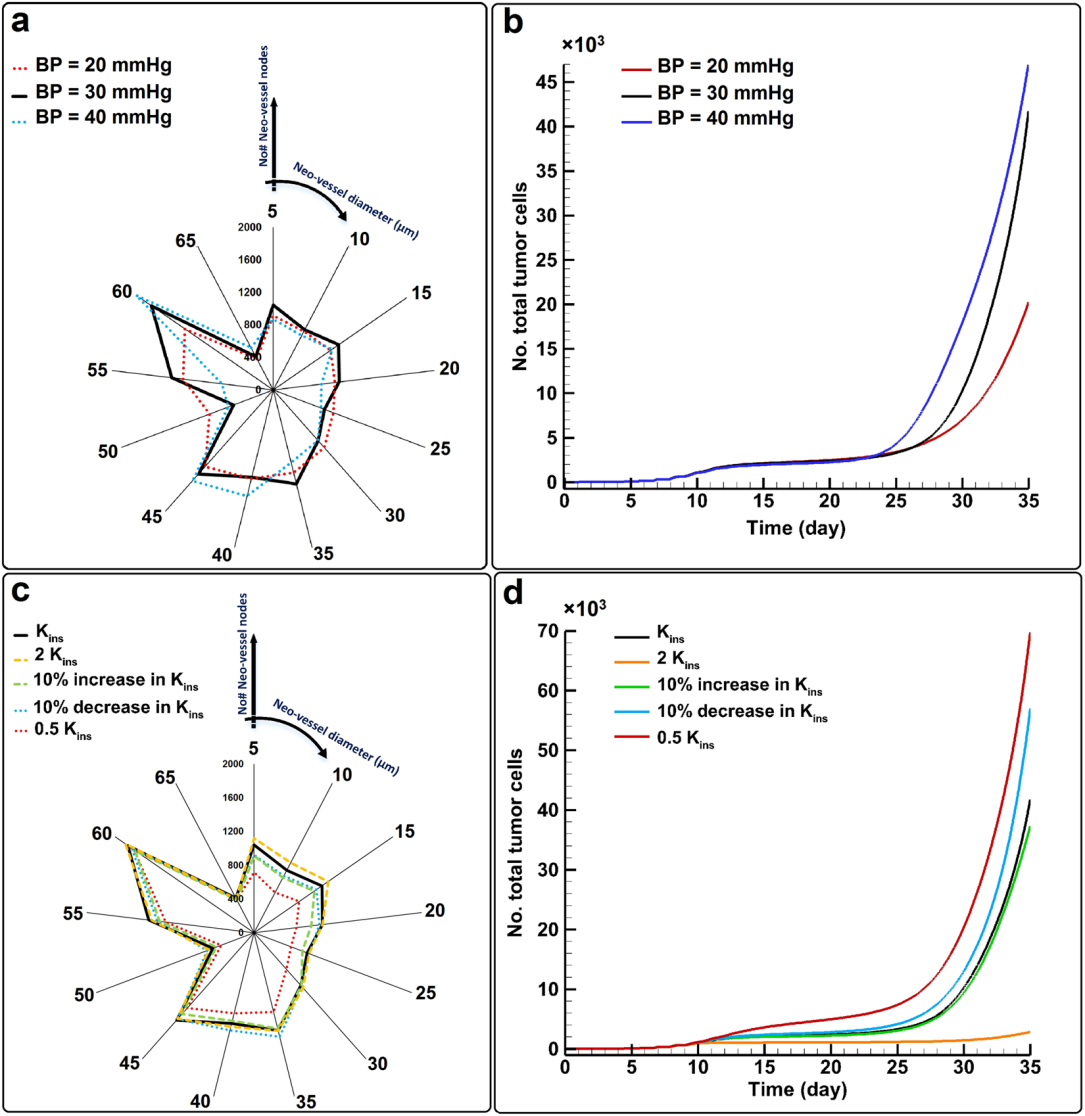
Effect of blood pressure and interstitial hydraulic conductivity (IHC) on tumor progression as two major biomechanical factors changing in response to chemotherapy treatments and desmoplasia tumor, respectively, (**a** and **c**) distribution of angiogenic neo-vessels, (**b** and **d**) total tumor cells (tumor size).

to further test the robustness of our model and its ability to predict a range of TME parameters, we next investigated the effect of blood pressure on tumor growth and angiogenesis by changing the inlet intravascular blood pressure from 30 mmHg to 20 and 40 mmHg (Fig. 8a,b). Increasing blood pressure skews the angiogenic neo-vessel distribution towards more large neo-vessels with diameters 55–65 µm (Fig. 8c) and significantly increases solid tumor size (Fig. 8b). Increased blood pressure is commonly encountered in cancer patients during chemotherapy treatment especially anti-angiogenesis drug treatment^94^. Therefore, there is a competition between drugs and drug-increased blood pressure to control tumor size.

**Figure 9:**
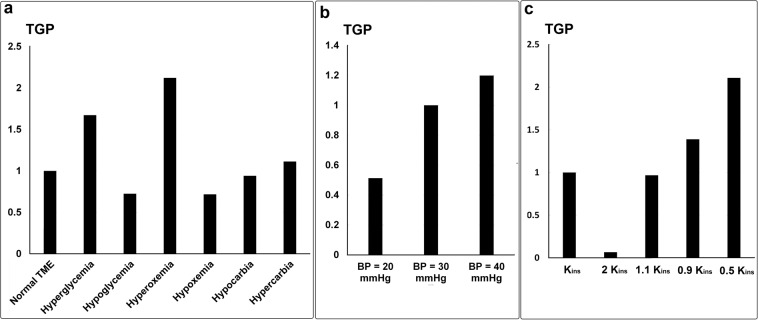
Tumor growth-performance (TGP) for parameter studies based on biochemical blood abnormalities (**a**), blood pressure (**b**), and interstitial hydraulic conductivity (**c**).

In another set of simulations, we varied IHC (K_ins_, see Eqs. 29 and 30) to see how tissue hydraulic conductivity might affect the tumor. We found that decreasing IHC results in fewer angiogenic neo-vessels and increased tumor size (total number of TCs; Fig. 8c,d). Consequently, this condition was most efficient in terms of tumor growth-performance (TGP), defined as the number of total TCs divided by the number of total angiogenic neo-vessels (see Fig. 9). Our results show that tumor growth is much more sensitive to IHC reduction than angiogenesis (Fig. 8c,d): The baseline IHC level and the abnormal cases with twice IHC, 10% increase and 10% decrease in IHC all have approximately the same vessel patterns (except a small difference in nascent and very small neo-vessels with diameters 5–15 µm). However, these different IHC levels produced completely different tumor sizes (Fig. 8d). Comparing tumor size at day 35, it is clear that decreasing the IHC of the TME can result in a bigger tumor, and there is a lag in growth if we increase IHC (Fig. 8d). These results highlight the importance of only one aspect of desmoplasia – IHC reduction^95^. However, desmoplasia involves other modifications to tissue structure, cell populations and biochemistry, so more detailed information and additional modeling is warranted.

Mechanistically, decreasing IHC increases interstitial fluid flow, in agreement with experimental measurements^95–98^. Increasing IFF velocity increases transport of oxygen, glucose and CO_2_, helping tumor cells to survive, proliferate and generate a larger mass. On the other hand, this would also help drug delivery, and a richer tumor with more oxygen concentration generates less VEGF, inducing fewer angiogenic neo-vessels (Fig. 8a).

We next compared the various blood chemistry, blood pressure and IHC conditions in terms of tumor growth-performance (TGP), defined as the number of TCs per angiogenic neo-vessel (Fig. 9). The results of various blood chemistry, blood pressure and IHC conditions have been normalized by the values of normal TME, BP = 30 mmHg, and K_ins_, respectively. For the biochemical blood abnormalities, the highest TGP was seen for hyperoxemia, while similar low values were produced by hypoxemia and hypoglycemia (Fig. 9a). Hyperglycemia also had a relatively high TGP, while hypercarbia and hypocarbia produced TGP values slightly higher and lower, respectively, than the normal condition. Increasing blood pressure increased TGP (Fig. 9b); therefore, high blood pressure can be a risk factor for cancer patients^94^. Finally, IHC had the most dramatic effect on TGP: the lowest and the highest TGP resulted from the highest IHC and lowest IHC, respectively (Fig. 9c). Another parameter study to show the effect of subcellular level components including ECM/MMPs, VEGF and angiopoietins on tumor growth performance has been presented in the Supplementary Material file.

## Validation of Tumor Growth and Angiogenesis Metrics and Trends

**Figure 10:**
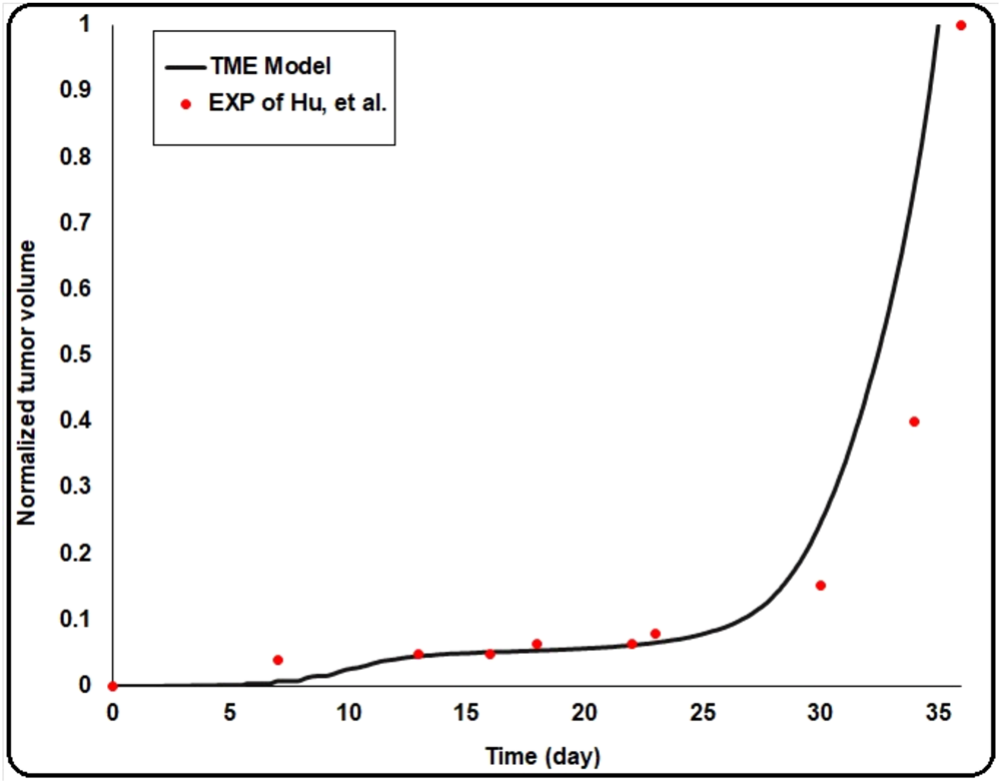
comparison of the TME model result for tumor growth with time and the experimental results of Hu, *et al*.^101^.

Some model predictions were compared and validated against the experimental studies, summarized in Table 2 as well as Fig. 10. The size of the avascular solid tumor before the onset of VTG and the three different phases of tumor growth are two common experimental observations evident in most tumor subtypes. According to Table 2, rows 1–3, and Fig. 10, the calculated results of our model are in good agreement with experimental measurements in terms of avascular solid tumor size and the dynamics of vascularization and vascularized growth.

**Table 2:** Experimental studies employed to compare and validate the present model predictions.

	Tumor Model	Parameters measured for model validation	Main experimental findings	Predictions of TME model
1	Colon cancer Human glioma tumor	Cancer cell populations	The tumor growth diagram has three different regions; 1) linear increment (for avascular tumor growth), 2) approximately constant size region (angiogenesis for converting from avascular to vascular tumor growth), and 3) exponentially increment region (for vascular tumor growth)^100,101^	Fig. 10: tumor volume with time
2	Iris tumor	relationship between angiogenesis and the ability of a tumor to grow malignantly	Solid tumor under avascular growth stops expanding at a very small size (less than 1 mm^3^), while the vascular tumor grows progressively exponentially^102^	Avascular solid tumor volume = 0.842 mm^3^
3	Most tumors	relationship between angiogenesis and the ability of a tumor to grow malignantly	Fig. 10a: Avascular tumors in the absence of angiogenesis can give rise to dormant microscopic tumors of ∼1 mm^3^ or less^30^	Avascular solid tumor volume = 0.842 mm^3^
4	Colon cancer	Effect of Hyperglycemia on tumor progression	Hyperglycemia increases cell proliferation and tumor progression (tumor weight increment to 5–110%)^99^	Hyperglycemia increases tumor volume to 53%
5	Breast cancer	Interstitial hydraulic conductivity	Desmoplasia can decrease interstitial hydraulic conductivity and then increase interstitial fluid pressure^95^	Decreasing interstitial hydraulic conductivity increases interstitial fluid pressure
6	Breast cancer	Tumor progression	Reduction of interstitial hydraulic conductivity results in increased tumor size (~28%)^95^	Reduction of interstitial hydraulic conductivity increases tumor size (25–57%)
7	Vascularized tumors	Vascular segment length	0.13–0.43 mm^103^ 0.06–0.3 mm^103^ 0.04–0.17 (0.104) mm^51^	0.05-0.56 (0.36) mm
8	Vascularized tumors	Bifurcation density	40.1–231.5 1/mm^3,103^ 97–918 1/mm^3,51^	60–650 1/mm^3^
9	Vascularized tumors	Angiogenic neo-vessel diameter	8–64 µm^103^	5–65 µm

Based on clinical studies, hyperglycemia is one of the important risk factors for cancer patients; it has been shown to increase tumor growth, causing larger tumors compared with control (5–110%) (Vasconcelos-Dos-Santos, *et al*.^99^). Our model reproduces this result, predicting a 53% increment in tumor size (Table 2, Row 4). Similarly, as mentioned above, desmoplasia has been shown to affect tumor growth and transport of nutrients and drugs. By decreasing interstitial hydraulic conductivity, desmoplasia increases interstitial fluid pressure (Mpekris, *et al*.^95^), and this increases tumor size by ~28%. We could reproduce the desmoplasia effects; theses simulations resulted in higher IFP and faster tumor growth with desmoplasia (size increased by 25–57%; Table 2, Row 5, Row 6).

The model also correctly predicts important vascular parameters such as vascular segment length, bifurcation density and diameter of angiogenic neo-vessels (Table 2, Rows 7–9).

## Conclusion

We presented a systems biology mathematical model of three-dimensional, multi-scale tumor microenvironment. We demonstrated the capabilities of the model by investigating how different TME conditions influence tumor progression during the various stages of tumor growth, including avascular tumor growth (ATG), angiogenesis, and vascular tumor growth (VTG). The model accounts for major biochemical and biomechanical factors in the TME, and these are all coupled across the subcellular, cellular, and tissue size scales. The model uses a comprehensive agent-based approach for the cell-signaling pathways in order to couple the various biological scales, creating a robust model able to recapitulate tumor progression under a variety of environmental conditions. We demonstrated the robustness and versatility of the model by simulating several common patient-specific TME disorders including biochemical blood abnormalities (hyperglycemia/hypoglycemia, hyperoxemia/hypoxemia, and hypercarbia/hypocarbia), increased blood pressure (an adverse event seen in chemotherapy patients) and altered interstitial hydraulic conductivity. The simulation results show that hyperoxemia/hyperglycemia blood abnormalities, chemotherapy-increased blood pressure, and reduction of interstitial hydraulic conductivity produce the most aggressive tumors with high tumor growth performance (TGP) values; these would be predicted to produce the worst patient outcome. All these blood abnormalities are possible in cancer patients, especially those receiving chemotherapy or radiotherapy treatments. We also elucidated the effects of subcellular level components including ECM, MMPs, VEGF and angiopoietins on tumor progression. The simulations show that the maximum TGP is produced when MMP level is low and VEGF secretion is high, and the minimum TGP results from high MMP secretion rates. One of the goals of developing this three-dimensional model was to accurately estimate the time delay before the onset of VTG (and thus the time of increased malignancy). Once the initial solid tumor is detected clinically, this can be determined under different environmental conditions using the model. This type of model is ideal for investigating the delivery and pharmacodynamics of anti-angiogenic and anti-cancer drugs, and their combinations to determine more effective treatment strategies to delay or prevent the switch to VTG, or to more effectively target solid tumors already in VTG. The model agrees well with experimental observations of tumor growth and angiogenesis, reproducing key features of angiogenesis, cancer cell metabolism and tumor asymmetric growth.

## Supplementary information

Supplementary material.

Three-dimensional tumor progression.

## Data Availability

The supporting data are availabe in the online supplemental files entitled ‘Supplementary Material’, and ‘Movie S1’. Source files of the TME model are accessible in Zenodo (DOI: 10.5281/zenodo.3609333).

## References

[1] Akkari, L. & Lujambio, A. In *Resistance to Molecular Therapies for Hepatocellular Carcinoma* 45–64 (Springer, 2017).

[2] Portillo-Lara R, Annabi N (2016). Microengineered cancer-on-a-chip platforms to study the metastatic microenvironment. Lab. a chip.

[3] Lee E, Song HG, Chen CS (2016). Biomimetic on-a-chip platforms for studying cancer metastasis. Curr. Opin. Chem. Eng..

[4] Shin Y (2011). *In vitro* 3D collective sprouting angiogenesis under orchestrated ANG-1 and VEGF gradients. Lab. a chip.

[5] Wood LB, Ge R, Kamm RD, Asada HH (2012). Nascent vessel elongation rate is inversely related to diameter in *in vitro* angiogenesis. Integr. Biol..

[6] Kim C, Kasuya J, Jeon J, Chung S, Kamm RD (2015). A quantitative microfluidic angiogenesis screen for studying anti-angiogenic therapeutic drugs. Lab. a chip.

[7] Song JW, Munn LL (2011). Fluid forces control endothelial sprouting. Proc. Natl Acad. Sci..

[8] Song JW, Bazou D, Munn LL (2012). Anastomosis of endothelial sprouts forms new vessels in a tissue analogue of angiogenesis. Integr. Biol..

[9] Song JW, Daubriac J, Janet MT, Bazou D, Munn LL (2012). RhoA mediates flow-induced endothelial sprouting in a 3-D tissue analogue of angiogenesis. Lab. a chip.

[10] Kashaninejad N (2016). Organ-tumor-on-a-chip for chemosensitivity assay: A critical review. Micromachines.

[11] Chen MB, Whisler JA, Jeon JS, Kamm RD (2013). Mechanisms of tumor cell extravasation in an *in vitro* microvascular network platform. Integr. Biol..

[12] Bersini S (2014). A microfluidic 3D *in vitro* model for specificity of breast cancer metastasis to bone. Biomater..

[13] Ehsan SM, Welch-Reardon KM, Waterman ML, Hughes CC, George SC (2014). A three-dimensional *in vitro* model of tumor cell intravasation. Integr. Biol..

[14] Chung S, Sudo R, Vickerman V, Zervantonakis IK, Kamm RD (2010). Microfluidic platforms for studies of angiogenesis, cell migration, and cell–cell interactions. Ann. Biomed. Eng..

[15] Skardal A, Devarasetty M, Rodman C, Atala A, Soker S (2015). Liver-tumor hybrid organoids for modeling tumor growth and drug response *in vitro*. Ann. Biomed. Eng..

[16] Shirure, V. S. *et al*. In *Tumor Organoids 117–148* (Springer, 2018).

[17] Cai Y, Xu S, Wu J, Long Q (2011). Coupled modelling of tumour angiogenesis, tumour growth and blood perfusion. J. Theor. Biol..

[18] Soltani M, Chen P (2013). Numerical modeling of interstitial fluid flow coupled with blood flow through a remodeled solid tumor microvascular network. PLoS one.

[19] Tang L (2014). Computational modeling of 3D tumor growth and angiogenesis for chemotherapy evaluation. PLoS one.

[20] Cai Y, Wu J, Li Z, Long Q (2016). Mathematical modelling of a brain tumour initiation and early development: a coupled model of glioblastoma growth, pre-existing vessel co-option, angiogenesis and blood perfusion. PLoS one.

[21] Stéphanou A (2017). How tumour-induced vascular changes alter angiogenesis: insights from a computational model. J. Theor. Biol..

[22] Vavourakis V (2017). A validated multiscale in-silico model for mechano-sensitive tumour angiogenesis and growth. PLoS computational Biol..

[23] Munn LL (2013). Dynamics of tissue topology during cancer invasion and metastasis. Phys. Biol..

[24] Jain RK, Tong RT, Munn LL (2007). Effect of vascular normalization by antiangiogenic therapy on interstitial hypertension, peritumor edema, and lymphatic metastasis: insights from a mathematical model. Cancer Res..

[25] Sun C, Jain RK, Munn LL (2007). Non-uniform plasma leakage affects local hematocrit and blood flow: implications for inflammation and tumor perfusion. Ann. Biomed. Eng..

[26] Nickerson NK (2012). Decreased autocrine EGFR signaling in metastatic breast cancer cells inhibits tumor growth in bone and mammary fat pad. PLoS one.

[27] Shieh AC, Swartz MA (2011). Regulation of tumor invasion by interstitial fluid flow. Phys. Biol..

[28] Cui X, Hartanto Y, Zhang H (2017). Advances in multicellular spheroids formation. J. R. Soc. Interface.

[29] Jiang Y, Pjesivac-Grbovic J, Cantrell C, Freyer JP (2005). A multiscale model for avascular tumor growth. Biophysical J..

[30] Folkman J (2006). Angiogenesis. Annu. Rev. Med..

[31] Anada T, Fukuda J, Sai Y, Suzuki O (2012). An oxygen-permeable spheroid culture system for the prevention of central hypoxia and necrosis of spheroids. Biomater..

[32] Munn, L. L., Kunert, C. & Tyrrell, J. A. In *Mathematical Methods and Models in Biomedicine* 117–147 (Springer, 2013).

[33] Wong BW, Marsch E, Treps L, Baes M, Carmeliet P (2017). Endothelial cell metabolism in health and disease: impact of hypoxia. EMBO J..

[34] Jakobsson L (2010). Endothelial cells dynamically compete for the tip cell position during angiogenic sprouting. Nat. Cell Biol..

[35] Wood L, Kamm R, Asada H (2011). Stochastic modeling and identification of emergent behaviors of an Endothelial Cell population in angiogenic pattern formation. Int. J. Robot. Res..

[36] Kim M-C, Silberberg YR, Abeyaratne R, Kamm RD, Asada HH (2018). Computational modeling of three-dimensional ECM-rigidity sensing to guide directed cell migration. Proc. Natl Acad. Sci..

[37] Deryugina EI, Quigley JP (2010). Pleiotropic roles of matrix metalloproteinases in tumor angiogenesis: contrasting, overlapping and compensatory functions. Biochimica et. Biophysica Acta -Molecular Cell Res..

[38] Gevertz JL, Torquato S (2006). Modeling the effects of vasculature evolution on early brain tumor growth. J. Theor. Biol..

[39] Baffert F (2004). Age-related changes in vascular endothelial growth factor dependency and angiopoietin-1-induced plasticity of adult blood vessels. Circulation Res..

[40] Carmeliet P (2003). Angiogenesis in health and disease. Nat. Med..

[41] Wang Z, Birch CM, Sagotsky J, Deisboeck TS (2009). Cross-scale, cross-pathway evaluation using an agent-based non-small cell lung cancer model. Bioinforma..

[42] Lesart A-C, Van Der Sanden B, Hamard L, Estève F, Stéphanou A (2012). On the importance of the submicrovascular network in a computational model of tumour growth. Microvascular Res..

[43] Shamsi M, Saghafian M, Dejam M, Sanati-Nezhad A (2018). Mathematical Modeling of the Function of Warburg Effect in Tumor Microenvironment. Sci. Rep..

[44] Xu J, Vilanova G, Gomez H (2016). A mathematical model coupling tumor growth and angiogenesis. PLoS one.

[45] Das A, Lauffenburger D, Asada H, Kamm RD (2010). A hybrid continuum–discrete modelling approach to predict and control angiogenesis: analysis of combinatorial growth factor and matrix effects on vessel-sprouting morphology. Philos. Trans. R. Soc. Lond. A: Mathematical, Phys. Eng. Sci..

[46] Chaturvedi R (2005). On multiscale approaches to three-dimensional modelling of morphogenesis. J. R. Soc. interface.

[47] Chaplain, M. & Anderson, A. In *Angiogenesis in Brain Tumors* 51–75 (Springer, 2004).

[48] Kashkooli, F. M., Soltani, M., Rezaeian, M., Taatizadeh, E. & Hamedi, M.-H. Image-based spatio-temporal model of drug delivery in a heterogeneous vasculature of a solid tumor—Computational approach. *Microvascular research* (2019). 10.1016/j.mvr.2019.01.005 30711547

[49] Voutouri, C. *et al*. Experimental and computational analyses reveal dynamics of tumor vessel cooption and optimal treatment strategies. *Proceedings of the National Academy of Sciences*, 201818322 (2019). 10.1073/pnas.1818322116 PMC6377457 30700544

[50] Zhao G (2007). Numerical simulation of blood flow and interstitial fluid pressure in solid tumor microcirculation based on tumor-induced angiogenesis. Acta Mechanica Sin..

[51] Norton K-A, Popel AS (2016). Effects of endothelial cell proliferation and migration rates in a computational model of sprouting angiogenesis. Sci. Rep..

[52] Welter M, Rieger H (2013). Interstitial fluid flow and drug delivery in vascularized tumors: a computational model. PLoS one.

[53] Anderson, A. R., Chaplain, M. A. & McDougall, S. In *Modeling Tumor Vasculature* 105–133 (Springer, 2012).

[54] Milde F, Bergdorf M, Koumoutsakos P (2008). A hybrid model for three-dimensional simulations of sprouting angiogenesis. Biophysical J..

[55] Cai Y, Zhang J, Li Z (2016). Multi-scale mathematical modelling of tumour growth and microenvironments in anti-angiogenic therapy. Biomed. Eng. online.

[56] Shirinifard A (2009). 3D multi-cell simulation of tumor growth and angiogenesis. PLoS one.

[57] Pries, A. R. & Secomb, T. W. Microvascular blood viscosity *in vivo* and the endothelial surface layer. *American Journal of Physiology-Heart and Circulatory Physiology* **289**, H2657-H2664 (2005). 10.1152/ajpheart.00297.2005 16040719

[58] Vander Heiden MG, Cantley LC, Thompson CB (2009). Understanding the Warburg effect: the metabolic requirements of cell proliferation. Sci..

[59] Ganapathy V, Thangaraju M, Prasad PD (2009). Nutrient transporters in cancer: relevance to Warburg hypothesis and beyond. Pharmacology therapeutics.

[60] Wheeler TJ (1986). Kinetics of glucose transport in human erythrocytes: zero-trans efflux and infinite-trans efflux at 0 C. Biochimica et. Biophysica Acta -Biomembranes.

[61] Berk, A., Zipursky, S. & Lodish, H. (National Center for Biotechnology InformationÕs Bookshelf, 2000).

[62] Buchwald P (2009). FEM-based oxygen consumption and cell viability models for avascular pancreatic islets. Theor. Biol. Med. Model..

[63] DeBerardinis RJ, Lum JJ, Hatzivassiliou G, Thompson CB (2008). The biology of cancer: metabolic reprogramming fuels cell growth and proliferation. Cell Metab..

[64] Skog S, Tribukait B, Sundius G (1982). Energy metabolism and ATP turnover time during the cell cycle of Ehrlich ascites tumour cells. Exp. Cell Res..

[65] del Toro R (2010). Identification and functional analysis of endothelial tip cell-enriched genes. Blood, blood.

[66] Bauer AL, Jackson TL, Jiang Y (2007). A Cell-Based Model Exhibiting Branching and Anastomosis during Tumor-Induced Angiogenesis. Biophysical J..

[67] Nikmaneshi M, Firoozabadi B, Saidi M (2018). Fully-coupled mathematical modeling of actomyosin-cytosolic two-phase flow in a highly deformable moving Keratocyte cell. J. Biomech..

[68] Nikmaneshi M, Firoozabadi B, Saidi M (2015). Two-Phase Acto-Cytosolic Fluid Flow in a Moving Keratocyte: A 2D Continuum Model. Bull. Math. Biol..

[69] Nikmaneshi, M. R., Firoozabadi, B. & Saidi, M. S. Continuum model of actin-myosin flow. In *2013 20th Iranian Conference on Biomedical Engineering (ICBME)*. 98–102 (IEEE).

[70] Nikmaneshi, M. R., Firoozabadi, B., Ghasemi, A. & Saidi, M. S. Development of mechanical stress in a moving cell: a continuum model.

[71] Baldwin ME (2001). The specificity of receptor binding by vascular endothelial growth factor-d is different in mouse and man. J. Biol. Chem..

[72] Seano G (2019). Solid stress in brain tumours causes neuronal loss and neurological dysfunction and can be reversed by lithium. Nat. Biomed. Eng..

[73] Nia HT (2016). Solid stress and elastic energy as measures of tumour mechanopathology. Nat. Biomed. Eng..

[74] Nia HT (2018). Quantifying solid stress and elastic energy from excised or *in situ* tumors. Nat. Protoc..

[75] Janet MT (2012). Mechanical compression drives cancer cells toward invasive phenotype. Proc. Natl Acad. Sci..

[76] Qazi H (2016). Heparan sulfate proteoglycans mediate renal carcinoma metastasis. Int. J. cancer.

[77] Polacheck WJ, Charest JL, Kamm RD (2011). Interstitial flow influences direction of tumor cell migration through competing mechanisms. Proc. Natl Acad. Sci..

[78] Pedersen JA, Lichter S, Swartz MA (2010). Cells in 3D matrices under interstitial flow: effects of extracellular matrix alignment on cell shear stress and drag forces. J. Biomech..

[79] Cameron, M. A. & Davis, A. L. A Mathematical Model of Angiogenesis in Glioblastoma Multiforme. (2009).

[80] Pries, A., Secomb, T. & Gaehtgens, P. Structural adaptation and stability of microvascular networks: theory and simulations. *American Journal of Physiology-Heart and Circulatory Physiology* **275**, H349-H360 (1998). 10.1152/ajpheart.1998.275.2.H349 9683420

[81] McDougall SR, Anderson AR, Chaplain MA (2006). Mathematical modelling of dynamic adaptive tumour-induced angiogenesis: clinical implications and therapeutic targeting strategies. J. Theor. Biol..

[82] Pries A, Reglin B, Secomb T (2001). Structural adaptation of microvascular networks: functional roles of adaptive responses. Am. J. Physiol.-Heart Circulatory Physiology.

[83] Stéphanou A, McDougall SR, Anderson AR, Chaplain MA (2006). Mathematical modelling of the influence of blood rheological properties upon adaptative tumour-induced angiogenesis. Math. Computer Model..

[84] Netti PA, Roberge S, Boucher Y, Baxter LT, Jain RK (1996). Effect of transvascular fluid exchange on pressure–flow relationship in tumors: a proposed mechanism for tumor blood flow heterogeneity. Microvascular Res..

[85] Baish JW, Netti PA, Jain RK (1997). Transmural coupling of fluid flow in microcirculatory network and interstitium in tumors. Microvascular Res..

[86] Welter M, Bartha K, Rieger H (2008). Emergent vascular network inhomogeneities and resulting blood flow patterns in a growing tumor. J. Theor. Biol..

[87] Roustaei M, Nikmaneshi MR, Firoozabadi B (2018). Simulation of Low Density Lipoprotein (LDL) permeation into multilayer coronary arterial wall: Interactive effects of wall shear stress and fluid-structure interaction in hypertension. J. Biomech..

[88] Roustaei, M., Nikmaneshi, M. R. & Firoozabadi, B. In *The 25th Annual International Conference on Mechanical Engineering ISME2017, COI: ISME25_041*.

[89] Chauhan VP (2014). Compression of pancreatic tumor blood vessels by hyaluronan is caused by solid stress and not interstitial fluid pressure. Cancer Cell.

[90] Alarcón T, Byrne HM, Maini PK (2003). A cellular automaton model for tumour growth in inhomogeneous environment. J. Theor. Biol..

[91] Roudnicky F (2018). Alternative transcription of a shorter, non-anti-angiogenic thrombospondin-2 variant in cancer-associated blood vessels. Oncogene.

[92] Vakoc BJ (2009). Three-dimensional microscopy of the tumor microenvironment *in vivo* using optical frequency domain imaging. Nat. Med..

[93] Hidrovo I (2017). Experimental method and statistical analysis to fit tumor growth model using SPECT/CT imaging: a preclinical study. Quant. imaging Med. Surg..

[94] Mouhayar E, Salahudeen A (2011). Hypertension in cancer patients. Tex. Heart Inst. J..

[95] Mpekris F (2017). Sonic-hedgehog pathway inhibition normalizes desmoplastic tumor microenvironment to improve chemo-and nanotherapy. J. Controlled Rel..

[96] Stylianopoulos T (2012). Causes, consequences, and remedies for growth-induced solid stress in murine and human tumors. Proc. Natl Acad. Sci..

[97] Stylianopoulos T, Munn LL, Jain RK (2018). Reengineering the physical microenvironment of tumors to improve drug delivery and efficacy: from mathematical modeling to bench to bedside. Trends cancer.

[98] Munn LL, Jain RK (2019). Vascular regulation of antitumor immunity. Sci..

[99] Vasconcelos-Dos-Santos A (2017). Hyperglycemia exacerbates colon cancer malignancy through hexosamine biosynthetic pathway. Oncogenesis.

[100] Prieto I (2017). Colon cancer modulation by a diabetic environment: A single institutional experience. PLoS one.

[101] Hu B (2007). Neuropilin-1 promotes human glioma progression through potentiating the activity of the HGF/SF autocrine pathway. Oncogene.

[102] Gimbrone MA, Leapman SB, Cotran RS, Folkman J (1972). Tumor dormancy *in vivo* by prevention of neovascularization. J. Exp. Med..

[103] Kim E (2012). Multiscale imaging and computational modeling of blood flow in the tumor vasculature. Ann. Biomed. Eng..

