# Supplementary material for: A multi-scale model for determining the effects of pathophysiology and metabolic disorders on tumor growth
Source: Sci Rep. 2020 Feb 20;10:3025. doi: 10.1038/s41598-020-59658-0 (PMC7033139; doi:10.1038/s41598-020-59658-0)
Supplement: Supplementary file 1 — Supplementary material. [file 41598_2020_59658_MOESM1_ESM.docx]

**Supplementary material for**

**A multi-scale model for determining the effects of pathophysiology and metabolic disorders on tumor growth**

**Mohammad R. Nikmaneshi ^1,2^, Aliasghar Mozafari^2^, Lance L. Munn^*1^, Bahar Firoozabadi^*2^**

**^1^Edwin L. Steele Laboratories, Department of Radiation Oncology, Massachusetts General Hospital, Harvard Medical School, Boston, MA 02114, USA.**

**^2^Department of Mechanical Engineering, Sharif University of Technology, Tehran, Iran**

**Correspondence and requests for materials should be addressed to L.L.M. (Email:** [**munn@steele.mgh.harvard.edu**](mailto:munn@steele.mgh.harvard.edu)**) or B.F. (Email:** [**firoozabadi@sharif.edu**](mailto:firoozabadi@sharif.edu)**).**

**This PDF file includes:**

Computational approach and model setup

Figs. S1 to S3

Table. S for Model Parameters

Biochemical and biomechanical distributions in the TME

Figs. S4 to S6

Effect of ECM/MMPs, VEGF, and angiopoietins on tumor growth and angiogenesis

Fig. S7

References for Supplementary material reference citations

**Computational approach and model setup**

To solve the governing equations in this model, we implement a double hybrid continuous-discrete (DHCD) method. In this computational approach, we separate the equations into two parts; 1) continuous part and 2) discrete part. The equations of the continuous part of the model are numerically solved with a finite difference mesh and an appropriate finite difference method (FDM). All the equations of the model except the angiogenesis and tumor growth equations (Eqs. (18) and (19) of paper) belong to the continuous part. Eqs. (18) and (19), as discrete parts of the model, are discretized on two distinct lattices with the same grids as the finite difference mesh. In the numerical chart presented in the next section, different parts of the model, the relationship between them as well as model equations solved in each part are shown.

**1) Continuous part of the DHCD:** the continuous part of the computational model consists of:

**i)** The biochemical equations, which govern the subcellular scale. These include: ADME equations of each CR-agent (oxygen, glucose and carbon dioxide, Eqs.1-5), VEGF and its receptor VEGFR-2 (Eqs.6-8), Ang-1 and Ang-2 and their receptor Tie-2 (Eqs.9-13), MMPs and ECM (Eqs. 14 and 15).

**ii)** The agent-based cellular vitality and energy equations, governing the cellular scale: Eqs.16 and 17.

**iii)** Vessel growth and remodeling at the tissue scale, including lumenogenesis (Eqs. 20 and 21), vessel adaptation (Eqs. 22a-22d and 23), and the vessel compliance effect (Eq. 24).

**iv)** Hemodynamics-interstitial fluid flow equations (Eqs. 25-30), and hemorheology equations (Eqs. 31-36), at the tissue scale.

Using an explicit forward difference scheme for time and a second-order central difference scheme for the space derivative at each position, we get an explicit method for solving the set of continuous parts of the equations. With the mesh shown in Fig. S1, the finite difference scheme around the central node (i, j, k) is: (i+1,j,k), (i-1,j,k), (i,j+1,k), (i,j-1,k), (i,j,k+1), (i,j,k-1).


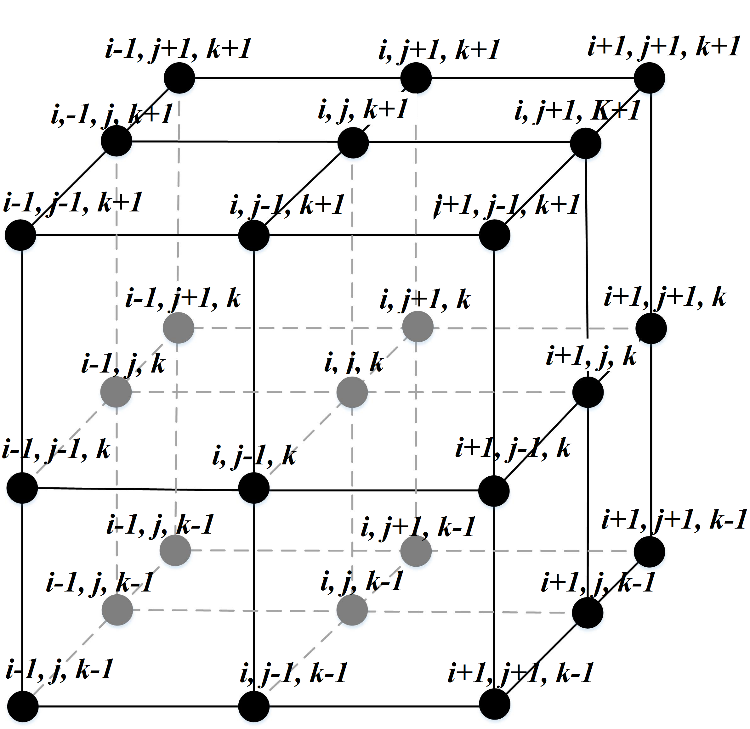


**Fig. S1. Computational finite difference mesh.**

**2) Discrete part of the DHCD:** The tumor growth and angiogenesis equations at the tissue scale (Eqs. 18 and 19) are discretized using a forward difference scheme in time and a second-order central difference scheme for the space derivative at position (FTCS). We consider all 26 neighborhood nodes shown in Fig. S2. Calculations use the probabilistic, discretized equations for tumor growth and angiogenesis (Eq. S1), which were derived for both endothelial cells (tECs) and tumor cells (TCs), which reside on two different lattices with the same grid size. At each time step, a given cell chooses the direction of motion based on the neighborhood probabilities and a generated random number. The computational lattice with an example motion probability distribution around the central cell is illustrated in Fig. S2. For simplicity, we select$\beta_{c}(c_{v})=\frac{\beta_{c}}{1+\alpha c_{v}}$ and$\lambda=\frac{\Delta t}{\Delta x^{2}}$. The probabilities of migration for a tEC and a TC are presented in Eqs. S2-28 and Eqs. S29-55, respectively. $c_{v}$and $c_{e}$in the migration probabilities equations (Eqs. S2-55) are calculated from the continuous part of the solution.


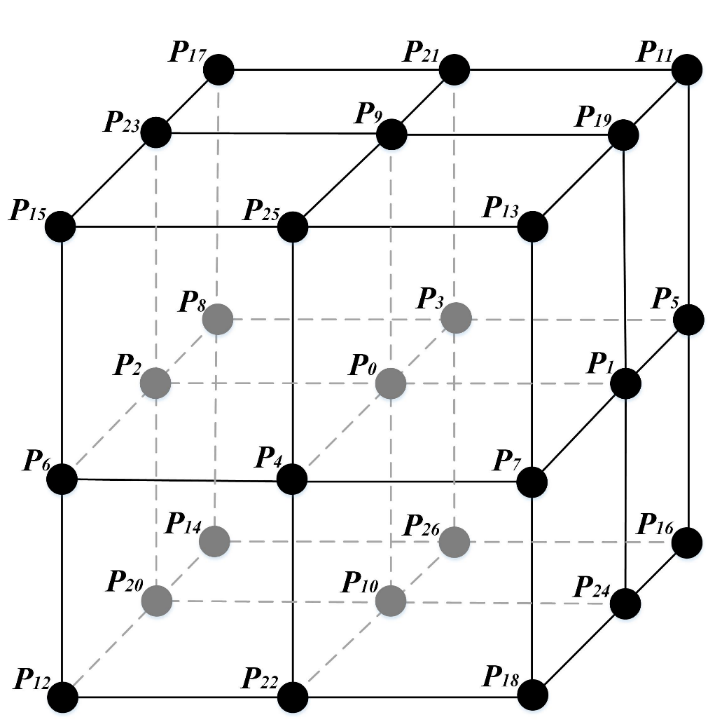


**Fig. S2. Computational lattice with spatial distribution of migration probabilities, *P_0-26_*, around a central cell (EC or TC).**

|  | (S1) |
| --- | --- |

**Migration probabilities for a central tEC:**

|  | (S2) |
| --- | --- |
| $P_{1} = \lambda D_{EC}+ \beta_{c}([c_{v}]_{ijk}^{n}) ([c_{v}]_{i+1jk}^{n}-[c_{v}]_{i-1jk}^{n})\lambda/4+\beta_{h} ([c_{e}]_{i+1jk}^{n}-[c_{e}]_{i-1jk}^{n})\lambda/4$ | (S3) |
| $P_{2} = \lambda D_{EC}- \beta_{c}([c_{v}]_{ijk}^{n}) ([c_{v}]_{i+1jk}^{n}-[c_{v}]_{i-1jk}^{n})\lambda/4-\beta_{h} ([c_{e}]_{i+1jk}^{n}-[c_{e}]_{i-1jk}^{n})\lambda/4$ | (S4) |
| $P_{3} = \lambda D_{EC}+ \beta_{c}([c_{v}]_{ijk}^{n}) ([c_{v}]_{ij+1k}^{n}-[c_{v}]_{ij-1k}^{n})\lambda/4+\beta_{h} ([c_{e}]_{ij+1k}^{n}-[c_{e}]_{ij-1k}^{n})\lambda/4$ | (S5) |
| $P_{4} = \lambda D_{EC}- \beta_{c}([c_{v}]_{ijk}^{n}) ([c_{v}]_{ij+1k}^{n}-[c_{v}]_{ij-1k}^{n})\lambda/4-\beta_{h} ([c_{e}]_{ij+1k}^{n}-[c_{e}]_{ij-1k}^{n})\lambda/4$ | (S6) |
| $P_{5} = \lambda/2 D_{EC}+ \beta_{c}([c_{v}]_{ijk}^{n}) ([c_{v}]_{i+1j+1k}^{n}-[c_{v}]_{i-1j-1k}^{n})\lambda/8+\beta_{h} ([c_{e}]_{i+1j+1k}^{n}-[c_{e}]_{i-1j-1k}^{n})\lambda/8$ | (S7) |
| $P_{6} = \lambda/2 D_{EC}-\beta_{c}([c_{v}]_{ijk}^{n}) ([c_{v}]_{i+1j+1k}^{n}-[c_{v}]_{i-1j-1k}^{n})\lambda/8-\beta_{h} ([c_{e}]_{i+1j+1k}^{n}-[c_{e}]_{i-1j-1k}^{n})\lambda/8$ | (S8) |
| $P_{7} = \lambda/2 D_{EC}+\beta_{c}([c_{v}]_{ijk}^{n}) ([c_{v}]_{i+1j-1k}^{n}-[c_{v}]_{i-1j+1k}^{n})\lambda/8+\beta_{h} ([c_{e}]_{i+1j-1k}^{n}-[c_{e}]_{i-1j+1k}^{n})\lambda/8$ | (S9) |
| $P_{8} = \lambda/2 D_{EC}-\beta_{c}([c_{v}]_{ijk}^{n}) ([c_{v}]_{i+1j-1k}^{n}-[c_{v}]_{i-1j+1k}^{n})\lambda/8-\beta_{h} ([c_{e}]_{i+1j-1k}^{n}-[c_{e}]_{i-1j+1k}^{n})\lambda/8$ | (S10) |
| $P_{9} = \lambda D_{EC}+\beta_{c}([c_{v}]_{ijk}^{n}) ([c_{v}]_{ijk+1}^{n}-[c_{v}]_{ijk-1}^{n})\lambda/4+\beta_{h} ([c_{e}]_{ijk+1}^{n}-[c_{e}]_{ijk-1}^{n})\lambda/4$ | (S11) |
| $P_{10} = \lambda D_{EC}-\beta_{c}([c_{v}]_{ijk}^{n}) ([c_{v}]_{ijk+1}^{n}-[c_{v}]_{ijk-1}^{n})\lambda/4-\beta_{h} ([c_{e}]_{ijk+1}^{n}-[c_{e}]_{ijk-1}^{n})\lambda/4$ | (S12) |
| $P_{11} = \lambda/3 D_{EC}+\beta_{c}([c_{v}]_{ijk}^{n}) ([c_{v}]_{i+1j+1k+1}^{n}-[c_{v}]_{i-1j-1k-1}^{n})\lambda/12+\beta_{h} ([c_{e}]_{i+1j+1k+1}^{n}-[c_{e}]_{i-1j-1k-1}^{n})\lambda/12$ | (S13) |
| $P_{12} = \lambda/3 D_{EC}-\beta_{c}([c_{v}]_{ijk}^{n}) ([c_{v}]_{i+1j+1k+1}^{n}-[c_{v}]_{i-1j-1k-1}^{n})\lambda/12-\beta_{h} ([c_{e}]_{i+1j+1k+1}^{n}-[c_{e}]_{i-1j-1k-1}^{n})\lambda/12$ | (S14) |
| $P_{13} = \lambda/3 D_{EC}+\beta_{c}([c_{v}]_{ijk}^{n}) ([c_{v}]_{i+1j-1k+1}^{n}-[c_{v}]_{i-1j+1k-1}^{n})\lambda/12+\beta_{h} ([c_{e}]_{i+1j-1k+1}^{n}-[c_{e}]_{i-1j+1k-1}^{n})\lambda/12$ | (S15) |
| $P_{14} = \lambda/3 D_{EC}-\beta_{c}([c_{v}]_{ijk}^{n}) ([c_{v}]_{i+1j-1k+1}^{n}-[c_{v}]_{i-1j+1k-1}^{n})\lambda/12-\beta_{h} ([c_{e}]_{i+1j-1k+1}^{n}-[c_{e}]_{i-1j+1k-1}^{n})\lambda/12$ | (S16) |
| $P_{15} = \lambda/3 D_{EC}+\beta_{c}([c_{v}]_{ijk}^{n}) ([c_{v}]_{i-1j-1k+1}^{n}-[c_{v}]_{i+1j+1k-1}^{n})\lambda/12+\beta_{h} ([c_{e}]_{i-1j-1k+1}^{n}-[c_{e}]_{i+1j+1k-1}^{n})\lambda/12$ | (S17) |
| $P_{16} = \lambda/3 D_{EC}-\beta_{c}([c_{v}]_{ijk}^{n}) ([c_{v}]_{i-1j-1k+1}^{n}-[c_{v}]_{i+1j+1k-1}^{n})\lambda/12-\beta_{h} ([c_{e}]_{i-1j-1k+1}^{n}-[c_{e}]_{i+1j+1k-1}^{n})\lambda/12$ | (S18) |
| $P_{17} = \lambda/3 D_{EC}+\beta_{c}([c_{v}]_{ijk}^{n}) ([c_{v}]_{i-1j+1k+1}^{n}-[c_{v}]_{i+1j-1k-1}^{n})\lambda/12+\beta_{h} ([c_{e}]_{i-1j+1k+1}^{n}-[c_{e}]_{i+1j-1k-1}^{n})\lambda/12$ | (S19) |
| $P_{18} = \lambda/3 D_{EC}-\beta_{c}([c_{v}]_{ijk}^{n}) ([c_{v}]_{i-1j+1k+1}^{n}-[c_{v}]_{i+1j-1k-1}^{n})\lambda/12-\beta_{h} ([c_{e}]_{i-1j+1k+1}^{n}-[c_{e}]_{i+1j-1k-1}^{n})\lambda/12$ | (S20) |
| $P_{19} = \lambda/2 D_{EC}+\beta_{c}([c_{v}]_{ijk}^{n}) ([c_{v}]_{i+1jk+1}^{n}-[c_{v}]_{i-1jk-1}^{n})\lambda/8+\beta_{h} ([c_{e}]_{i+1jk+1}^{n}-[c_{e}]_{i-1jk-1}^{n})\lambda/8$ | (S21) |
| $P_{20} = \lambda/2 D_{EC}-\beta_{c}([c_{v}]_{ijk}^{n}) ([c_{v}]_{i+1jk+1}^{n}-[c_{v}]_{i-1jk-1}^{n})\lambda/8-\beta_{h} ([c_{e}]_{i+1jk+1}^{n}-[c_{e}]_{i-1jk-1}^{n})\lambda/8$ | (S22) |
| $P_{21} = \lambda/2 D_{EC}+\beta_{c}([c_{v}]_{ijk}^{n}) ([c_{v}]_{ij+1k+1}^{n}-[c_{v}]_{ij-1k-1}^{n})\lambda/8+\beta_{h} ([c_{e}]_{ij+1k+1}^{n}-[c_{e}]_{ij-1k-1}^{n})\lambda/8$ | (S23) |
| $P_{22} = \lambda/2 D_{EC}-\beta_{c}([c_{v}]_{ijk}^{n}) ([c_{v}]_{ij+1k+1}^{n}-[c_{v}]_{ij-1k-1}^{n})\lambda/8-\beta_{h} ([c_{e}]_{ij+1k+1}^{n}-[c_{e}]_{ij-1k-1}^{n})\lambda/8$ | (S24) |
| $P_{23} = \lambda/2 D_{EC}+\beta_{c}([c_{v}]_{ijk}^{n}) ([c_{v}]_{i-1jk+1}^{n}-[c_{v}]_{i+1jk-1}^{n})\lambda/8+\beta_{h} ([c_{e}]_{i-1jk+1}^{n}-[c_{e}]_{i+1jk-1}^{n})\lambda/8$ | (S25) |
| $P_{24} = \lambda/2 D_{EC}-\beta_{c}([c_{v}]_{ijk}^{n}) ([c_{v}]_{i-1jk+1}^{n}-[c_{v}]_{i+1jk-1}^{n})\lambda/8-\beta_{h} ([c_{e}]_{i-1jk+1}^{n}-[c_{e}]_{i+1jk-1}^{n})\lambda/8$ | (S26) |
| $P_{25} = \lambda/2 D_{EC}+\beta_{c}([c_{v}]_{ijk}^{n}) ([c_{v}]_{ij-1k+1}^{n}-[c_{v}]_{ij+1k-1}^{n})\lambda/8+\beta_{h} ([c_{e}]_{ij-1k+1}^{n}-[c_{e}]_{ij+1k-1}^{n})\lambda/8$ | (S27) |
| $P_{26} = \lambda/2 D_{EC}-\beta_{c}([c_{v}]_{ijk}^{n}) ([c_{v}]_{ij-1k+1}^{n}-[c_{v}]_{ij+1k-1}^{n})\lambda/8-\beta_{h} ([c_{e}]_{ij-1k+1}^{n}-[c_{e}]_{ij+1k-1}^{n})\lambda/8$ | (S28) |

**Migration probabilities for a central TC:**

|  | | (S29) | |
| --- | --- | --- | --- |
| $P_{1} = \lambda D_{EC}+\beta_{h} ([c_{e}]_{i+1jk}^{n}-[c_{e}]_{i-1jk}^{n})\lambda/4$ | | (S30) | |
| $P_{2} = \lambda D_{EC}-\beta_{h} ([c_{e}]_{i+1jk}^{n}-[c_{e}]_{i-1jk}^{n})\lambda/4$ | | (S31) | |
| $P_{3} = \lambda D_{EC}+\beta_{h} ([c_{e}]_{ij+1k}^{n}-[c_{e}]_{ij-1k}^{n})\lambda/4$ | | (S32) | |
| $P_{4} = \lambda D_{EC}-\beta_{h} ([c_{e}]_{ij+1k}^{n}-[c_{e}]_{ij-1k}^{n})\lambda/4$ | | (S33) | |
| $P_{5} = \lambda/2 D_{EC}+\beta_{h} ([c_{e}]_{i+1j+1k}^{n}-[c_{e}]_{i-1j-1k}^{n})\lambda/8$ | | (S34) | |
| $P_{6} = \lambda/2 D_{EC}-\beta_{h} ([c_{e}]_{i+1j+1k}^{n}-[c_{e}]_{i-1j-1k}^{n})\lambda/8$ | | (S35) | |
| $P_{7} = \lambda/2 D_{EC}+\beta_{h} ([c_{e}]_{i+1j-1k}^{n}-[c_{e}]_{i-1j+1k}^{n})\lambda/8$ | | (S36) | |
| $P_{8} = \lambda/2 D_{EC}-\beta_{h} ([c_{e}]_{i+1j-1k}^{n}-[c_{e}]_{i-1j+1k}^{n})\lambda/8$ | | (S37) | |
| $P_{9} = \lambda D_{EC}+\beta_{h} ([c_{e}]_{ijk+1}^{n}-[c_{e}]_{ijk-1}^{n})\lambda/4$ | | (S38) | |
| $P_{10} = \lambda D_{EC}-\beta_{h} ([c_{e}]_{ijk+1}^{n}-[c_{e}]_{ijk-1}^{n})\lambda/4$ | | (S39) | |
| $P_{11} = \lambda/3 D_{EC}+\beta_{h} ([c_{e}]_{i+1j+1k+1}^{n}-[c_{e}]_{i-1j-1k-1}^{n})\lambda/12$ | | (S40) | |
| $P_{12} = \lambda/3 D_{EC}-\beta_{h} ([c_{e}]_{i+1j+1k+1}^{n}-[c_{e}]_{i-1j-1k-1}^{n})\lambda/12$ | | (S41) | |
| $P_{13} = \lambda/3 D_{EC}+\beta_{h} ([c_{e}]_{i+1j-1k+1}^{n}-[c_{e}]_{i-1j+1k-1}^{n})\lambda/12$ | | (S42) | | |
| $P_{14} = \lambda/3 D_{EC}-\beta_{h} ([c_{e}]_{i+1j-1k+1}^{n}-[c_{e}]_{i-1j+1k-1}^{n})\lambda/12$ | | (S43) | |
| $P_{15} = \lambda/3 D_{EC}+\beta_{h} ([c_{e}]_{i-1j-1k+1}^{n}-[c_{e}]_{i+1j+1k-1}^{n})\lambda/12$ | | (S44) | |
| $P_{16} = \lambda/3 D_{EC}-\beta_{h} ([c_{e}]_{i-1j-1k+1}^{n}-[c_{e}]_{i+1j+1k-1}^{n})\lambda/12$ | | (S45) | |
| $P_{17} = \lambda/3 D_{EC}+\beta_{h} ([c_{e}]_{i-1j+1k+1}^{n}-[c_{e}]_{i+1j-1k-1}^{n})\lambda/12$ | | (S46) | |
| $P_{18} = \lambda/3 D_{EC}-\beta_{h} ([c_{e}]_{i-1j+1k+1}^{n}-[c_{e}]_{i+1j-1k-1}^{n})\lambda/12$ | | (S47) | |
| $P_{19} = \lambda/2 D_{EC}+\beta_{h} ([c_{e}]_{i+1jk+1}^{n}-[c_{e}]_{i-1jk-1}^{n})\lambda/8$ | | (S48) | |
| $P_{20} = \lambda/2 D_{EC}-\beta_{h} ([c_{e}]_{i+1jk+1}^{n}-[c_{e}]_{i-1jk-1}^{n})\lambda/8$ | | (S49) | |
| $P_{21} = \lambda/2 D_{EC}+\beta_{h} ([c_{e}]_{ij+1k+1}^{n}-[c_{e}]_{ij-1k-1}^{n})\lambda/8$ | | (S50) | |
| $P_{22} = \lambda/2 D_{EC}-\beta_{h} ([c_{e}]_{ij+1k+1}^{n}-[c_{e}]_{ij-1k-1}^{n})\lambda/8$ | | (S51) | |
| $P_{23} = \lambda/2 D_{EC}+\beta_{h} ([c_{e}]_{i-1jk+1}^{n}-[c_{e}]_{i+1jk-1}^{n})\lambda/8$ | | (S52) | |
| $P_{24} = \lambda/2 D_{EC}-\beta_{h} ([c_{e}]_{i-1jk+1}^{n}-[c_{e}]_{i+1jk-1}^{n})\lambda/8$ | | (S53) | |
| $P_{25} = \lambda/2 D_{EC}+\beta_{h} ([c_{e}]_{ij-1k+1}^{n}-[c_{e}]_{ij+1k-1}^{n})\lambda/8$ | | (S54) | |
| $P_{26} = \lambda/2 D_{EC}-\beta_{h} ([c_{e}]_{ij-1k+1}^{n}-[c_{e}]_{ij+1k-1}^{n})\lambda/8$ | | (S55) | |
|  |  | |

The diagram of computational strategies for the model in each time step is presented in Fig. S3. This chart shows different compartments for each length scale of the model and the relationships between these compartments, as well as the related equations solved to update each compartment. The continuous and discrete parts of the model are respectively solved by FDM (on the finite difference mesh, Fig. S1, with discretized equations of the continuous parts) and lattice (on the lattice shown in Fig. S2 with the set of probabilities presented in Eqs. S2-S55).

***Model setup steps:***

**1.** Set initial and boundary conditions,

**2.** Update subcellular agents on finite difference mesh, Fig.S1:

- Update O_2_, glucose, and CO_2_ fields using Eq. (1-3) coupled by set of (Eqs. (4), (5a-c), (16)), Eqs. (18) and (19) for L_EC_ and L_TC_, and Eqs. (25-36) for convection by u_ins_.
- Update ECM and MMPs fields using the set of coupled Eqs. (14) and (15) coupled with Eq. (16) for cellular vitality-dependent production term of MMPs, Eqs. (18) and (19) for LEC and LTC, and Eqs. (25-36) for convection by u_ins_.
- Update VEGF and VEGFR-2 fields using the set of coupled Eqs. (6-8) coupled with Eq. (1) for O2 dependent production term of VEGF, Eqs. (18) and (19) for L_EC_ and L_TC_, and Eqs. (25-36) for convection by u_ins_.
- Update Ang-1, Ang-2 and Tie-2 fields using the set of coupled Eqs. (9-13) coupled with Eqs. (18) and (19) for L_EC_ and L_TC_, and Eqs. (25-36) for convection by u_ins_.

**3.** Update cellular scale on finite difference mesh, Fig.S1:

- Update cellular vitality (𝝑) field using Eq. (16) coupled with Eqs. (1), (2), (3) of step 2.
- Update cellular energy (ψ) field using Eq. (17) coupled with Eqs. (16).
- Update tumor cell and endothelial phenotypes

**4.** Update tissue scale on finite difference mesh, Fig.S1, for hemodynamics, interstitial fluid flow, tumor-induced solid stress, and vessel growth and remodeling variables:

- Vessel growth and remodeling: a) Lumenogenesis using the set of coupled Eqs. (20) and (21) coupled with Eqs. (6), (9), and (10), Eqs. (18) for L_EC_. b) Vessel adaptation using the set of coupled Eqs. (22a-d) and (23) coupled with WSS, transvascular pressure, hematocrit, and VEGF. c) Vessel deformation using Eq. (24) coupled with solid pressure and transvascular pressure, d) Vessel disruption based on WSS and VEGF.
- Update Hemodynamics parameters: a) Update intravascular blood pressure by integrating Eqs. (25-27), iteratively solved and updated by changing all vessel structure and diameter values, vessel wall hydraulic conductivity, oncotic pressure, and hemorheology, b) Update other hemodynamics parameters (WSS, velocity and flowrate) using Hagen-Poiseuille’s law and calculated blood flow pressure.
- Update Interstitial fluid flow: a) Update interstitial fluid pressure by solving Eq. (30), iteratively solved and updated by changing all vessel structure and diameter values, vessel wall and tissue hydraulic conductivity, oncotic pressure, and hemorheology, b) Update interstitial fluid velocity by solving Eq. (29), using Darcy’s law and calculated interstitial fluid pressure.
- Update hemorheology (blood viscosity) using the set of coupled Eqs. (31-36).
- Update the interstitium and vessel properties for tumor and stroma, and update tumor-induced solid pressure field.

**5.** Update tissue scale on lattice of tumor cells, Fig.S2, for tumor growth: Tumor growth using Eq. (19) coupled with Eq. (14) for haptotaxis, and checking empty TCs spaces weighted by low solid pressure, and high nutrient.

**6.** Update tissue scale on lattice of endothelia cells, Fig.S2, for angiogenesis: Angiogenesis using Eq. (18) coupled with Eq. (14) for haptotaxis and Eq. (6) for chemotaxis, and checking empty ECs spaces weighted by low solid pressure.

**7.** Update the subcellular and then cellular scales based on the updated tissue scale information.

**
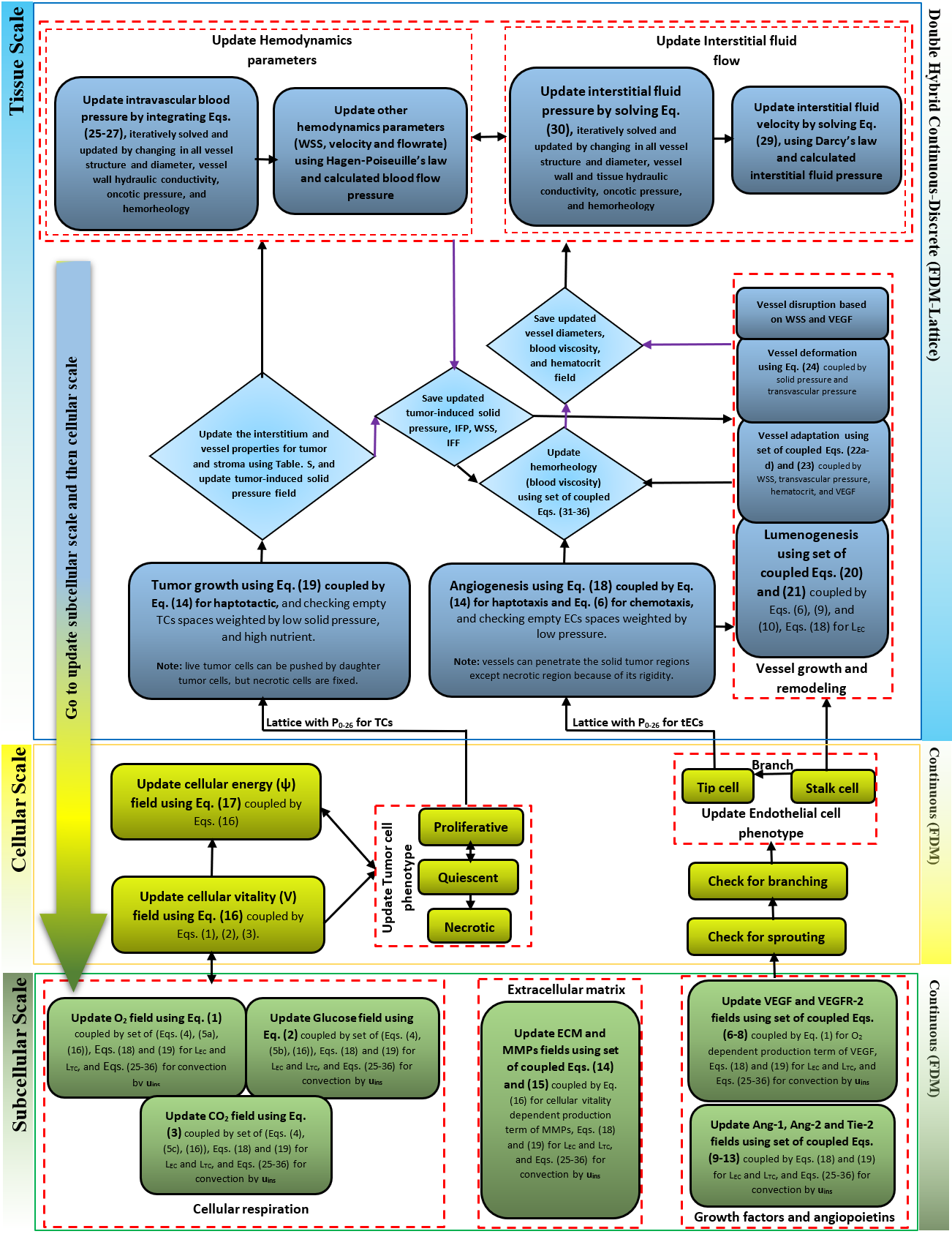
**

**Fig. S3. Schematic flow chart of the TME model to show the model setup and relationships between calculation steps.**

**Model Parameters**

**Table. S. The parameters used for computational results of the mathematical model.**

| **Parameters** | **Description** | **Value** | **References** |
| --- | --- | --- | --- |
| Cellular respiration | | | |
| $D_{o_{2}}$ | Diffusion coefficient of oxygen | 8×10^-14^ m^2^/s | Wang and Li ^1^ Estimated |
| $D_{g}$ | Diffusion coefficient of glucose | 8×10^-14^ m^2^/s | Estimated |
| $D_{co_{2}}$ | Diffusion coefficient of carbon-dioxide | 4×10^-14^ m^2^/s | Tang, et al. ^2^ Estimated |
| $\gamma_{0}$ | Maximum consumption/production rate of CR-agents | 3×10^-8^ M/s | Tang, et al. ^2^ |
| $f_{o_{2}}$ | Efflux rate of oxygen from RBCs | 6.8×10^-7^ M/s | Wang and Li ^1^ |
| $f_{g}$ | Maximum efflux rate of glucose from RBCs | 8×10^-6^ M/s | Wheeler ^3^ |
| $km_{g}$ | M-M constant of glucose perfusion | 3.4×10^-3^ M | Wheeler ^3^ |
| $f_{co_{2}}$ | Constant transport rate of carbon-dioxide | 2.5×10^-5^ 1/s | Tang, et al. ^2^ |
| $c_{o_{2}}^{ch}$ | Characteristic oxygen concentration | 8.4×10^-3^ M | Tang, et al. ^2^ |
| $c_{g}^{ch}$ | Characteristic glucose concentration | 8.7×10^-3^ M | Wheeler ^3^ |
| $c_{co_{2}}^{ch}$ | Characteristic carbon-dioxide concentration | 10.5×10^-3^ M | Tang, et al. ^2^ |
| Growth factors | | | |
| $D_{v}$ | Diffusion coefficient of VEGF | 1.2×10^-13^ m^2^/s | Anderson and Chaplain ^4^,  Tang, et al. ^2^ Estimated |
| $f_{v}$ | VEGF influx rate into vessels | Very small →0 1/s | Tang, et al. ^2^ |
| $R_{v}$ | VEGF production rate by TCs | 2×10^-12^ M/s | Tang, et al. ^2^ |
| $k_{v}^{+}$ | Binding rate of VEGF by VEGFR-2 | 1.3×10^-2^ 1/(µM.s) | Baldwin, et al. ^5^ |
| $k_{v}^{-}$ | Unbinding rate of VEGF from VEGFR-2 | 6.3×10^-5^ 1/s | Baldwin, et al. ^5^ |
| $\varepsilon_{v}$ | Natural excretion rate of VEGF | 2.78×10^-7^ 1/s | Gevertz and Torquato ^6^ |
| $c_{v}^{ch}$ | Characteristic VEGF concentration | 1.1×10^-8^ M | Tang, et al. ^2^ |
| Angiopoietins | | | |
| $D_{a1}$ | Diffusion coefficient of Ang-1 | Very small →0 m^2^/s | Gevertz and Torquato ^6^ |
| $D_{a2}$ | Diffusion coefficient of Ang-2 | 1×10^-13^ m^2^/s | Gevertz and Torquato ^6^ Estimated |
| $R_{a1}$ | Secretion rate of Ang-1 by ECs | 2.78×10^-6^ 1/s | Gevertz and Torquato ^6^ |
| $R_{a2}$ | Secretion rate of Ang-2 by ECs associated with tumor tissue | 2.22×10^-5^ 1/s | Gevertz and Torquato ^6^ |
| $k_{a1}^{+}$ | Binding rate of Ang-1 to Tie-2 | 1×10^-2^ 1/(µM.s) | Davis, et al. ^7^  Longstaff ^8^ |
| $k_{a1}^{-}$ | Unbinding rate of Ang-1 from Tie-2 | 3.7×10^-5^ 1/s | Davis, et al. ^7^  Longstaff ^8^ |
| $k_{a2}^{+}$ | Binding rate of Ang-2 to Tie-2 | 1.16×10^-2^ 1/(µM.s) | Maisonpierre, et al. ^9^  Gevertz and Torquato ^6^ |
| $k_{a2}^{-}$ | Unbinding rate of Ang-2 from Tie-2 | 3×10^-5^ 1/s | Maisonpierre, et al. ^9^  Gevertz and Torquato ^6^ |
| $K_{a}$ | Carrying capacity coefficient of angiopoietins | 1.5×10^-2^ µM | Gevertz and Torquato ^6^ |
| $e_{0}$ | Characteristic concentration of ECs at each blood vessel | 1×10^-4^ µM | Plank, et al. ^10^ |
| $\varepsilon_{a1}$ | Natural excretion rates of Ang-1 | 8.33×10^-7^ 1/s | Gevertz and Torquato ^6^ |
| $\varepsilon_{a2}$ | Natural excretion rates of Ang-2 | 5.56×10^-7^ 1/s | Gevertz and Torquato ^6^ |
| Extracellular matrix | | | |
| $D_{m}$ | Diffusion coefficient of MMPs | 1×10^-13^ m^2^/s | Cai, et al. ^11^ |
| $R_{m,T}$ | Secretion rates of MMPs by TCs | 1.7×10^-13^ M/s | Estimated |
| $R_{m,E}$ | Secretion rates of MMPs by ECs | 0.3×10^-13^ M/s | Estimated |
| $\varepsilon_{m}$ | Natural excretion rates of MMPs | 1.7×10^-8^ 1/s | Cai, et al. ^11^ |
| $\varepsilon_{e}$ | Natural excretion rates of ECM | 1.3×10^-7^ 1/s | Cai, et al. ^11^ |
| $c_{e}^{ch}$ | Characteristic ECM concentration | 1.36×10^-9^ M | Estimated |
| $c_{m}^{ch}$ | Characteristic MMP concentration | 1.36×10^-9^ M | Estimated |
| Cellular vitality and energy | | | |
|  | Proportionality coefficient of consumption/production rate of CR-agents | 3.67 | Buchwald ^12^ |
| $V^{ch}$ | Characteristic cellular vitality for active TCs | 0.5 | Estimated |
| $\psi^{ch}$ | Characteristic cellular energy for proliferation | 30 | Estimated |
| $k_{q}^{c}$ | Constant consumption rate of cellular energy by quiescent TCs | 0.1 | Tang, et al. ^2^ |
| $k_{a}^{p}$ | Coefficient of production rate of cellular energy by active TCs | 1 | Estimated |
| $k_{a}^{c}$ | Maximum consumption rate of cellular energy by active TCs | 1 | Estimated |
| Solid Tumor growth and Angiogenesis | | | |
| $D_{EC}$ | Diffusivity of ECs | 1×10^-13^ m^2^/s | Cai, et al. ^11^ |
| $D_{TC}$ | Diffusivity of TCs | 1×10^-13^ m^2^/s | Cai, et al. ^11^ |
| $\beta_{c}$ | Weight coefficient of chemotaxis | 0.26 m^2^/(M.s) | Cai, et al. ^11^ |
| $\beta_{h}$ | Weight coefficient of haptotaxis | 0.1 m^2^/(M.s) | Cai, et al. ^11^ |
| $\alpha$ | Saturation coefficient of chemotaxis | 1 | Cai, et al. ^11^ |
| Vessel growth and remodeling | | | |
| $G_{0}$ | M-M constant of neo-vessel lumen growth | 500 | Tang, et al. ^2^ |
| $\alpha_{p}$ | Maximum rate of sEC proliferation | 0.198 1/h | Cameron and Davis ^13^ |
| $\theta_{p}$ | M-M constant of sEC proliferation | 2.8×10^-6^ mol VEGF/ 1 m^3^ EC | Cameron and Davis ^13^ |
| $\omega_{m}$ | Maximum quiescent persistence rate of sEC | 0.576 1/h | Cameron and Davis ^13^ |
| $\theta_{m}$ | M-M constant of sEC proliferation relative to VEGF | 1.6×10^-4^ mol VEGF/ 1 m^3^ EC | Cameron and Davis ^13^ |
| $\rho_{m}$ | M-M constant of sEC proliferation relative to Ang-1/Ang-2 | 1 | Cameron and Davis ^13^ |
| $\delta$ | Maximum rate of sECs death | 0.198 1/h | Cameron and Davis ^13^ |
| $\theta_{d}$ | M-M constant of sECs death | 3.3×10^-7^ mol VEGF/ 1 m^3^ EC | Cameron and Davis ^13^ |
| $k_{age}$ | constant neo-vessel lumen growth due to aging | 1.4 1/h | Tang, et al. ^2^ |
| $Q_{ref}$ | Maximum flow rate of the neo-vessel network | 1.909×10^-11^ | Stéphanou, et al. ^14^ |
| $\tau_{ref}$ | A positive constant as reference of WSS | 7.73×10^-5^ mmHg | Pries, et al. ^15^ |
|  | Characteristic elastic stress to ensure stability | 1 mmHg | Stéphanou, et al. ^16^ |
| $k_{p}$ | Proportional coefficient of transvascular stimuli | 0.5 1/s | Stéphanou, et al. ^14^ |
| $k_{m}$ | Proportional coefficient of metabolic stimuli | 0.12 1/s | Stéphanou, et al. ^14^ |
| $k_{e}$ | Proportional coefficient of VEGF-dependent elastic stress | 5 1/s | Stéphanou, et al. ^16^ |
| $S_{Sh}$ | Inherent tendency of vessels to shrink and decrease their diameter | 0.35 | Stéphanou, et al. ^14^ |
| $E$ | Constant elasticity of neo-vessels | 6.5 mmHg | Netti, et al. ^17^ |
| $cp$ | Compliance power of neo-vessels | 0.19 | Netti, et al. ^17^ |
| $p_{c}$ | Collapse pressure of neo-vessels | 3 mmHg | Netti, et al. ^17^ |
| $\tau_{th}$ | Threshold endothelial WSS concentration for vessel disruption | 0.03 mmHg | Stéphanou, et al. ^16^ |
| $c_{v}^{th}$ | Threshold VEGF concentration for vessel disruption | 8 times$c_{v}^{ch}$ | Estimated based on Stéphanou, et al. ^16^ |
| $t_{s}$ | Survival time for vessel disruption under low WSS and high VEGF | 60 h | Stéphanou, et al. ^16^ |
| $d_{c}$ | Characteristic diameter of neo-vessels | 50 µm | Estimated |
| $d_{0}$ | Stress-free vessel diameter | 30 µm | Stéphanou, et al. ^16^ |
| Hemodynamics-interstitial fluid flow of TME | | | |
| $K_{ins}$ | Interstitial hydraulic conductivity (IHC) of TME | 9×10^-15^m2/(mmHg.s) for healthy tissue, and 4.5×10^-15^m2/(mmHg.s) for tumor tissue | Estimated |
| $L_{p}$ | Hydraulic conductivity of angiogenic neo-vessels wall | Constant value for healthy tissue, 3.6×10^-10^ m/(mmHg.s)  Maximum value for tumor tissue, 28×10^-10^ m/(mmHg.s) | Cai, et al. ^18^, Cai, et al. ^19^ |
| $\sigma$ | Osmotic reflection coefficient | 0.91 for healthy tissue and 0.82 for tumor tissue | Soltani and Chen ^20^, Zhao, et al. ^21^ |
| $\pi_{lum}$ | collide osmotic (oncotic) pressures of intravascular plasma | 20 mmHg for both healthy and tumor tissues | Soltani and Chen ^20^, Zhao, et al. ^21^ |
| $\pi_{ins}$ | collide osmotic (oncotic) pressures of interstitial fluid | 10 mmHg for heathy tissue and 15 mmHg for tumor tissue | Soltani and Chen ^20^, Zhao, et al. ^21^ |
| $S/V$ | Characteristic value of surface area of neo-vessels per unit volume for mass transport in the interstitium | 7×10^3^m^-1^ for healthy tissue and 2×10^4^m^-1^ for tumor tissue | Soltani and Chen ^20^, Zhao, et al. ^21^ |
| $u_{0}$ | Characteristic TME velocity | 5×10^-6^ m/s | Estimated |
| $u_{0,f}$ | Characteristic flow velocity | 1.3×10^-2^ m/s | Estimated |
| $p_{0}$ | Characteristic TME pressure and WSS | 60 mmHg | Tang, et al. ^2^ |
| Hemorheology | | | |
| $r_{th}$ | Threshold blood velocity ratio of two branches in bifurcations | 2.8 | Alarcón, et al. ^22^, Soltani and Chen ^20^ |
| $H_{D}^{n}$ | Normal hematocrit of healthy blood | 0.45 | Pries and Secomb ^23^ |
| $\mu_{plasma}$ | Plasma dynamic viscosity | 9×10^-6^ mmHg.s | Pries and Secomb ^23^ |

**Biochemical and biomechanical distributions in the TME**

***Biochemical agents of the TME:*** Spatiotemporal distributions of non-dimensional concentrations of ECM and biochemical agents in the TME (including MMPs, VEGF, Ang-1&-2) that can either directly or indirectly influence the ECM during tumor progression are shown in Fig. S4 for the middle cross-section of the 3D domain. VEGF, Ang-1 and -2 are non-denationalized by $c_{v}^{ch}$, ECM is non-denationalized by $c_{e}^{ch}$, and MMP is non-denationalized by $c_{m}^{ch}$. TCs and ECs secret MMPs to degrade the ECM and create available spaces for their migration. As shown in Fig. S4 of MMP-day 35, MMP concentration correlates well with different regions of the tumor: the necrotic-core has low concentration, the quiescent-regions have intermediate concentration, and the active-region has a high concentration, located just inside the tumor periphery.

The VEGF concentration increases during avascular tumor growth (ATG) and angiogenesis (ATG-to-VTG; days 10-20), and immediately decreases after the arrival of the angiogenic neo-vessels at the tumor periphery, as these vessels bring more oxygen to the TCs (days 25-30). During vascular tumor growth (VTG; days 30-35), due to reduction of oxygen inside the tumor – especially regions occupied by living cells – VEGF concentration increases again. VEGF concentration inside the tumor is also affected by binding to and unbinding from VEGFR-2 receptors expressed by tumor associated ECs (shown in Fig. S5) ^6^. Ang-1 is responsible for maturation and stabilization of immature vessels by promoting EC survival, and is constitutively expressed by ECs (Fig. S4 of Ang-1). After penetration of vessels into the tumor tissue, Ang-2, which destabilizes vessels through promoting EC death, begins to be expressed by ECs (Fig. S4). Indeed, since Ang-1 and Ang-2 share the same receptor Tie-2, Ang-2 destabilizes vessels by competing with Ang-1 for binding to Tie-2.


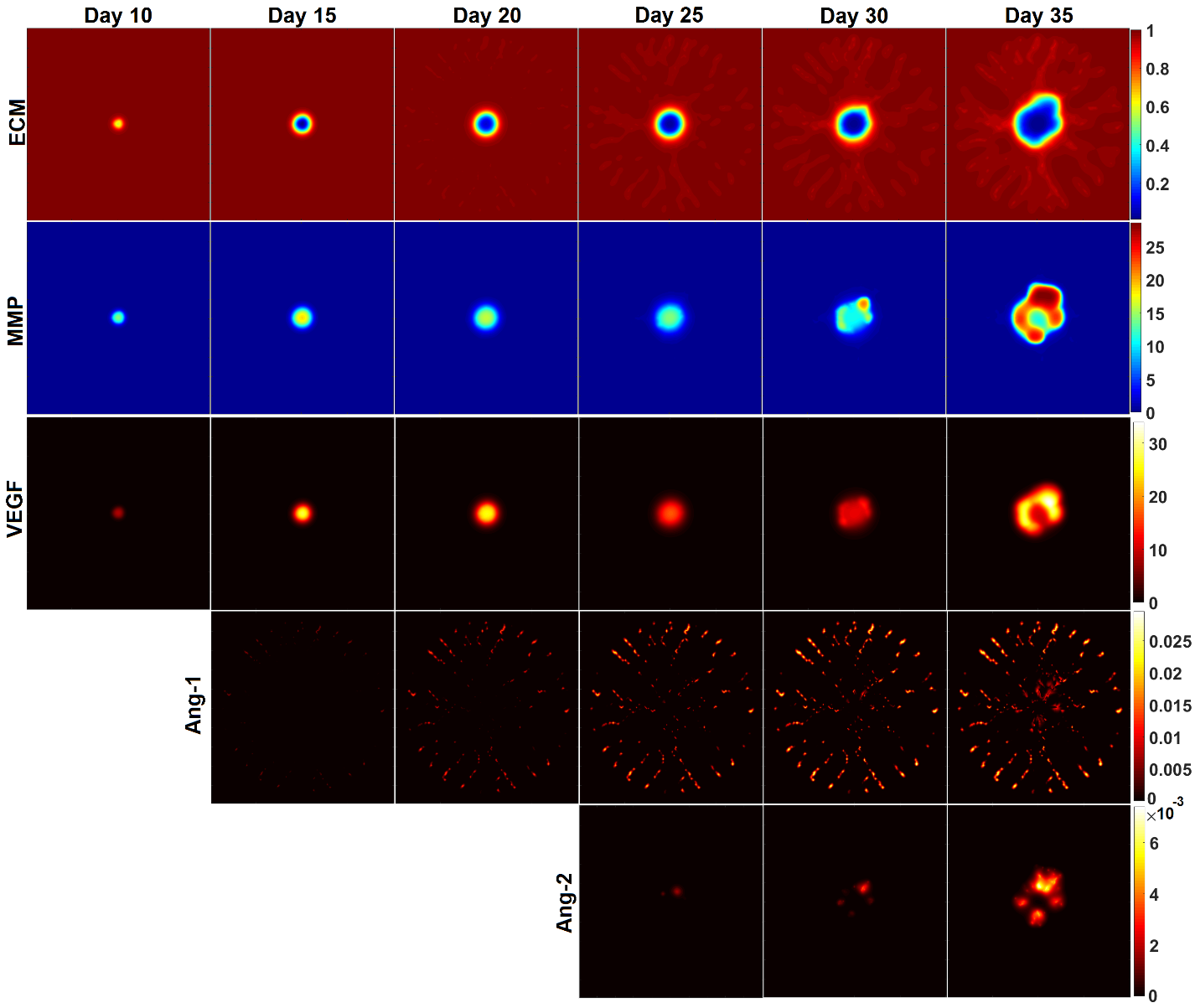


**Figure S4. Distributions of ECM, MMP, VEGF, Ang-1 and Ang-2 in the middle cross-section of 3D domain during days 10, 15, 20, 25, 35 of tumor progression.**

Distributions of non-dimensional concentrations of unbound free-Tie-2, active-Tie-2 bound-by-Ang-1 (Tie-2/Ang-1) and bound-by-Ang-2 (Tie-2/Ang-2), unbound free-VEFGR-2 and active-VEFGR-2 bound-by-VEGF (VEGFR-2/VEGF) are shown in Fig. S5. The values of these parameters are non-denationalized by $c_{v}^{ch}$. As can be seen, unbound Tie-2 and Tie-2/Ang-1 are expressed throughout the angiogenesis process, but complexes of Tie-2/Ang-2 and VEGFR-2/VEGF, and unbound free-VEGFR-2 are expressed only when neo-vessels penetrate the tumor during VTG. Because of the competition between Ang-1 )neo-vessel stabilization factor( and Ang-2) neo-vessel destabilization factor( for binding to their common receptor, Tie-2, the high concentration regions of Tie-2/Ang-2 during VTG (days 30-35) represent regions where neo-vessels are being destabilized.


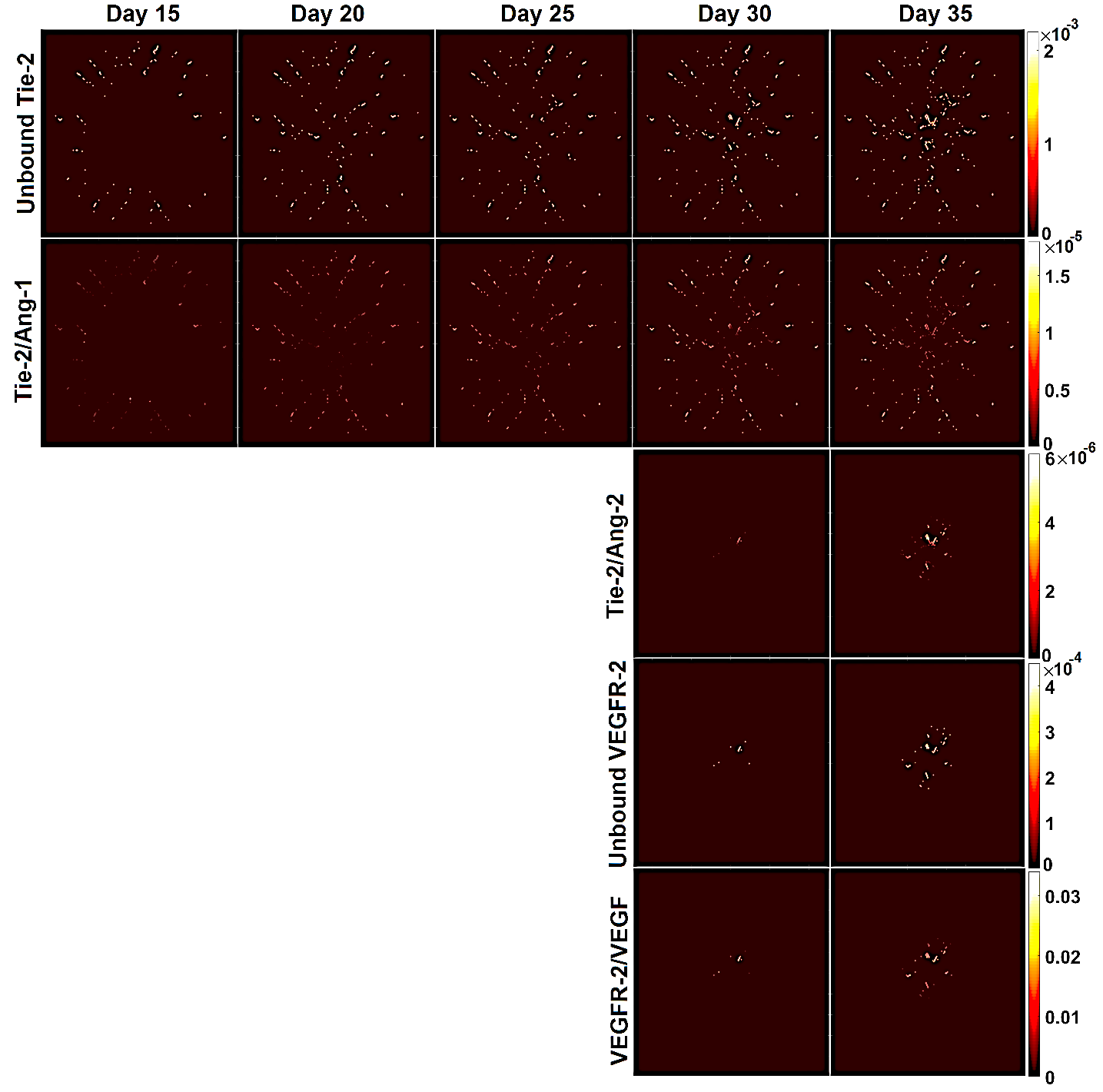


**Figure S5. Distributions of unbound Tie-2, Tie-2/Ang-1, Tie-2/Ang-2, unbound VEGFR-2, and VEGFR-2/VEGF in the middle cross-section of 3D domain during days 10, 15, 20, 25, 35 of tumor progression.**

***Biomechanical factors in the TME:*** Non-dimensional TME pressure ($p_{ins} + p_{s}$), interstitial fluid flow (IFF) velocity, intravascular blood pressure and WSS for vascularized tumor are shown in Fig. S6. The pressures (intravascular blood pressure and TME pressure) and WSS are normalized by $p_{0}$ and IFF velocity is normalized by $u_{0,f}$ (see Table S). Vessel perfusion and aggregates of TCs have been considered as two sources of mechanical pressure. For a vascularized tumor, association of very leaky tumor neo-vessels to TCs, significantly increases TME pressure in the tumor peripheral regions, Fig. S6 of TME pressure. Tumor-induced evolutions include: 1) the increased intratumoral neo-vessel hydraulic conductivity, 2) the increased vessel area-per-volume for transport into the interstitium, 3) the higher oncotic pressure of the tumor, and 4) the reduction of the tumor’s hydraulic conductivity are responsible for amplifying IFP (see Table S). High mechanical pressure gradient in the periphery region of vascularized tumor drives a high velocity IFF, Fig. S6 of u_ins_. The calculated distributions of TME pressure and IFF are in agreement with the other studies that have investigated the pressure and fluid flows within solid tumors ^11,20,24,25^.

According to Fig. S6, P_lum_, there is high intravascular blood pressure in the older matured neo-vessels with larger diameters, but it drops in the younger neo-vessels, which have smaller diameters and are mostly located around the necrotic core of tumor. The WSS distribution also has the same trend as that of intravascular blood pressure. Therefore, older matured neo-vessels are exposed to more intense WSS- and pressure-induced stimuli than younger immature/nascent neo-vessels. On the other hand, younger vessels located in the high VEGF regions would experience more intense VEGF-induced stimulus. Vessel remodeling in response to hematocrit follows the same pattern as the WSS-induced stimulus, and is also increased in the matured older neo-vessels.


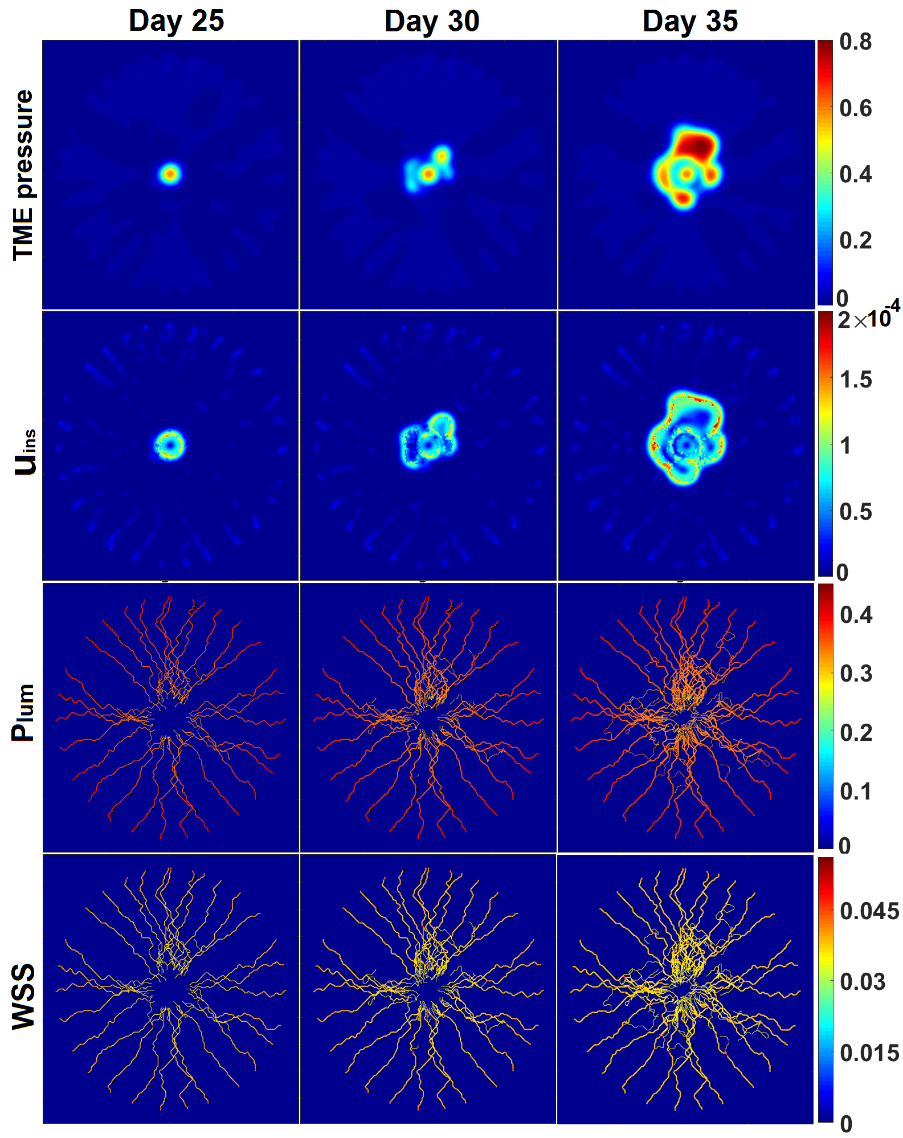


**Figure S6. Distributions of interstitial pressure, interstitial fluid flow velocity, intravascular pressure, and WSS in the middle cross-section of the 3D domain during days 25,30, and 35 of tumor progression. 3D neo-vessels have been projected onto the 2D plane at the middle cross-section.**

**Effect of ECM/MMPs, VEGF, and angiopoietins on tumor growth and angiogenesis**

We also elucidated the effects of ECM, MMPs, VEGF and angiopoietins on tumor morphology by changing MMP secretion rates of both ECs and TCs, VEGF secretion rate of TCs, and the amounts of ang-1/ang-2 driving the lumenogenesis process, respectively. The results of tumor growth performance (TGP), angiogenic neo-vessels and tumor growth shown in Fig. S7 have been normalized by the values of the normal TME. In this study, high and low rates mean 10 and 0.1 times the normal rates, respectively. Increasing MMP secretion rate means increasing the ECM degradation rate (Eqs. 14 and 15 of main text) which also generates a high adhesion (fibronectin) gradient around each cell, and thus intensifies haptotactic migration of cells. A result of intensified haptotaxis is an increase in the total number of angiogenic neo-vessels. On the other hand, a high concentration of MMPs can decrease the tumor volume by degrading the ECM. Increasing the secretion rate of VEGF, which is a main agent of angiogenesis, increases neo-vessel number and thus intensifies the delivery of nutrients, so the tumor volume is increased. Increasing ang-1/ang-2 increases the quiescence persistence rate (Eq. (21) of main text), which reduces the diameters of angiogenic neo-vessels as well as their nutrient supply efficacy. In response to low nutrients (especially oxygen) and consequently high VEGF concentration (VEGF production term of Eq.7 of main text) neo-vessels start to increase their branching, increasing the total number of neo-vessels. Indeed, in the case of high/low ang-1/ang-2, there is a competition between the increase or decrease of vessel branching and the reduction or increase of neo-vessel diameter to compensate for the reduced nutrient supply. For high ang-1/ang-2, the negative effect of neo-vessel diameter reduction on tumor volume is dominant compared to the positive effect of increased neo-vessel branching, and for low ang-1/ang-2, the negative effect of reduced neo-vessel branching is dominant compared to the positive effect of increased neo-vessel diameter. Therefore, both cases reduce the tumor volume. As a numerical conclusion for this parameter study, the maximum TGP belongs to low MMPs and high VEGF secretion rates, and the minimum TGP is for high MMP secretion rates.


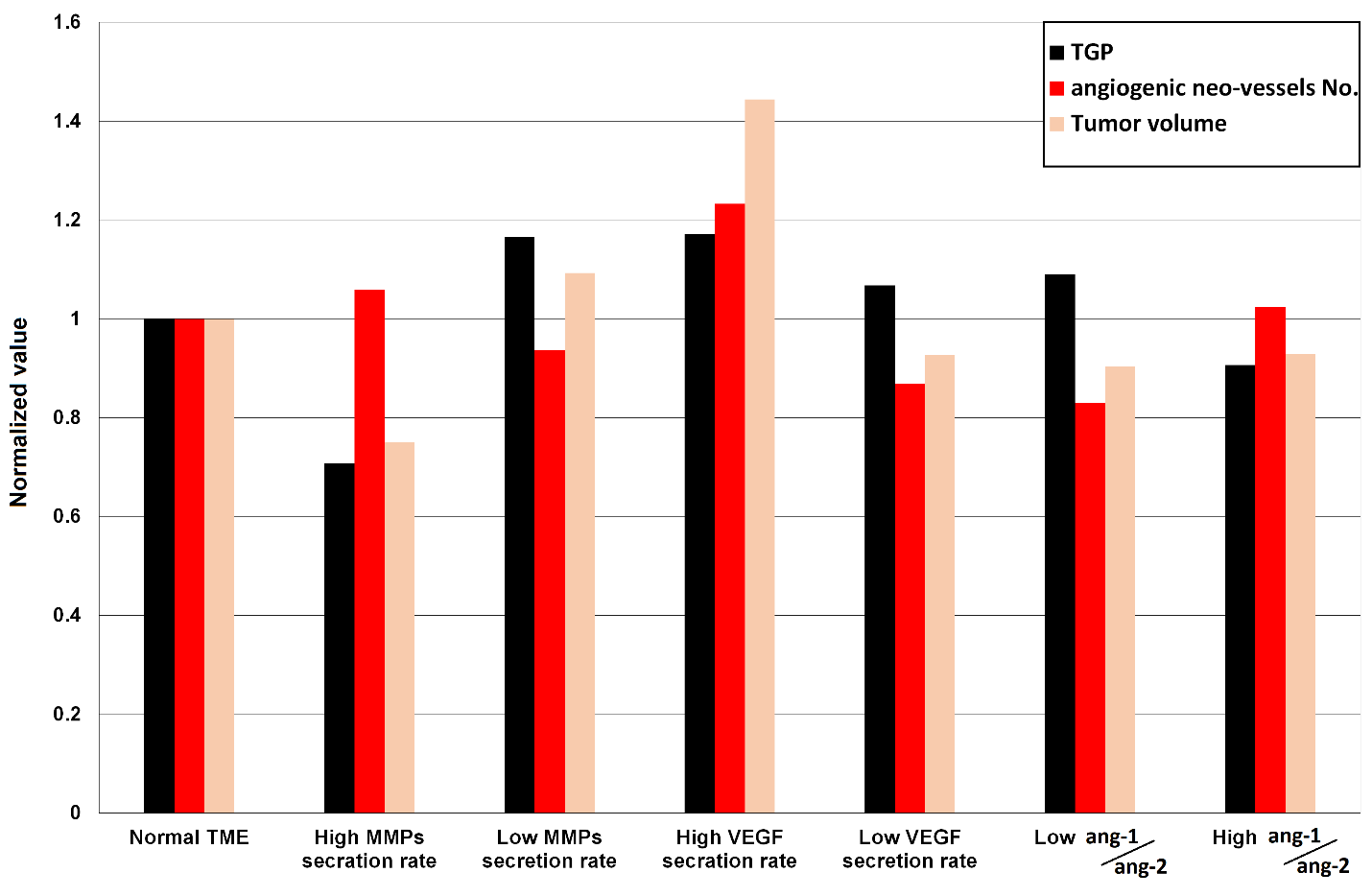


**Figure S7. Effects of ECM/MMPs interaction, VEGF, and angiopoietins on TGP, angiogenic neo-vessels and tumor volume.**

Table.S2 summarizes previous 2D mathematical models of the tumor microenvironment relevant to this work.

**Table. S2. Two-dimensional Mathematical models of the tumor microenvironment and angiogenesis**

|  | **Domain** | | **Tumor**  **Growth** | | |  |  | **Cellular**  **Level** | | **Angiogenesis Vessels** | | | | | **Fluid Dynamics** | | |  |  |
| --- | --- | --- | --- | --- | --- | --- | --- | --- | --- | --- | --- | --- | --- | --- | --- | --- | --- | --- | --- |
| **Tumor**  **Tissue**  Ref | **Blood**  **Vessels** | **Avascular**  **Growth** | **Angio-**  **genesis** | **Vascular Growth** |  | **Model**  **Formulation** | **Tumor Cells**  **Subcellular (Molecular level)** | **Endothelial Cells** | **Lumeno-genesis** | **Vessel Adaptation** | **Vessel Deformation** | **Branching** |  | **Hemo-rheology** | **Hemo-dynamics**  **Disruption** | **Interstitial**  **Fluid Flow** | **Solid**  **Mechanics** |  | **Notes** |
| 2D models |  |  |  |  |  |  |  |  |  |  |  |  |  |  |  |  |  |  |  |
| Zhao, et al. ^49^ | **-** | **+** | **-** | **+** | **-** | **Discrete** | **TAF, FN** | **-** | **+,a** | **-** | **-** | **-** | **+,b** | **-** | **-** | **+** | **+** | **-** | ^a^TAF gradient-induced chemotaxis and fibronectin gradient-induced haptotaxis; ^b^Based on sprout age and endothelia cell density |
| Soltani and Chen ^17^ | **-** | **+** | **-** | **+** | **-** | **Discrete** | **-** | **-** | **-** | **-** | **+,a** | **-** | **+,b** | **-** | **+,c** | **+** | **+** | **-** | ^a^Depends on WSS, intravascular pressure; metabolism based on hematocrit; ^b^Stochastic; ^c^ Vessel diameter and hematocrit affect blood viscosity; bifurcations affect hematocrit |
| Cai, et al. ^16^ | **+** | **+** | **-** | **+** | **+** | **HCD** | **O_2_, FN, MMP, VEGF** | **+.a** | **+,b** | **-** | **+,c** | **+** | **+,d** | **+,e** | **+,f** | **+** | **+** | **-** | ^a^Cell phenotype depends on O_2_; ^b^TAF gradient-induced chemotaxis and fibronectin gradient-induced haptotaxis; ^c^Depends on WSS, Intravascular pressure, Metabolic mechanism based on hematocrit; ^d^Based on age of sprouts and vessel density; ^e^Based on WSS; ^f^Viscosity depends on hematocrit and vessel diameter |
| Stéphanou, et al. ^20^ | **+** | **+** | **+** | **+** | **+** | **HCD** | **O_2_, FN, MMP, VEGF** | **+.a** | **+,b** | **-** | **+,c** | **-** | **+,d** | **+,e** | **-** | **-** | **-** | **-** | ^a^Cell phenotype depends on O_2_; ^b^TAF gradient-induced chemotaxis and fibronectin gradient-induced haptotaxis; ^c^Depends on WSS; VEGF (VEGF-dependent susceptibility to adaptation); Intravascular pressure; metabolism based on hematocrit; ^d^Stochastic; ^e^Based on WSS and VEGF |
| Lesart, et al. ^41^ | **+** | **+** | **+** | **+** | **+** | **HCD** | **O_2_, FN, MMP, VEGF** | **+,a** | **+,b** | **-** | **-** | **-** | **+, c** | **+, d** | **+,e** | **+** | **+** | **-** | ^a^Cell phenotype depends on O_2_; ^b^TAF gradient-induced chemotaxis and fibronectin gradient-induced haptotaxis; ^c^ Stochastic; ^d^ Depends on WSS; ^e^Viscosity depends on hematocrit and vessel diameter; |
| Shamsi, et al. ^42^ | **+** | **+** | **+** | **+** | **+** | **HCD** | **O_2_, glucose, VEGF, H+** | **+,a** | **+,b** | **-** | **-** | **-** | **+,c** | **-** | **+,d** | **+** | **+** | **-** | ^a^Cell phenotype depends on O_2_ and glucose; ^b^TAF gradient-induced chemotaxis and fibronectin gradient-induced haptotaxis; ^c^Based on sprout age and endothelia cell density; ^d^Viscosity depends on hematocrit |
| Kashkooli, et al. ^47^ | **-** | **+,a** | **-** | **-** | **-** | **Continuous** | **-** | **-** | **-** | **-** | **-** | **-** | **-** | **-** | **-** | **+** | **+** | **-** | ^a^Static vessel network |
| Voutouri, et al. ^48^ | **+** | **+** | **-** | **-** | **-** | **Continuous** | **VEGF, Ang-1, Ang-2, SDF1a, PDGF-B** | **+,a** | **+,b** | **-** | **-** | **-** | **-** | **-** | **-** | **-** | **-** | **-** | ^a^O_2_ and SDF1a affect tumor cell migration and proliferation; ^b^VEGF-induced chemotactic for endothelial migration |
| Xu, et al. ^43^ | **+** | **+** | **+** | **+** | **+** | **Continuous** | **TAF, A nutrient** | **+,a** | **+,b** | **-** | **-** | **-** | **+,c** | **-** | **-** | **-** | **-** | **-** | ^a^A nutrient determines tumor cell proliferation; ^b^VEGF-induced chemotaxis for endothelial migration; ^c^Stochastic |

References

1 Wang, C.-H. & Li, J. Three-dimensional simulation of IgG delivery to tumors. *Chemical Engineering Science* **53**, 3579-3600 (1998).

2 Tang, L. *et al.* Computational modeling of 3D tumor growth and angiogenesis for chemotherapy evaluation. *PloS one* **9**, e83962 (2014).

3 Wheeler, T. J. Kinetics of glucose transport in human erythrocytes: zero-trans efflux and infinite-trans efflux at 0 C. *Biochimica et Biophysica Acta (BBA)-Biomembranes* **862**, 387-398 (1986).

4 Anderson, A. R. & Chaplain, M. Continuous and discrete mathematical models of tumor-induced angiogenesis. *Bulletin of mathematical biology* **60**, 857-899 (1998).

5 Baldwin, M. E. *et al.* The specificity of receptor binding by vascular endothelial growth factor-d is different in mouse and man. *Journal of Biological Chemistry* **276**, 19166-19171 (2001).

6 Gevertz, J. L. & Torquato, S. Modeling the effects of vasculature evolution on early brain tumor growth. *Journal of Theoretical Biology* **243**, 517-531 (2006).

7 Davis, S. *et al.* Isolation of angiopoietin-1, a ligand for the TIE2 receptor, by secretion-trap expression cloning. *Cell* **87**, 1161-1169 (1996).

8 Longstaff, C. Plasminogen activation on the cell surface. *Front. Biosci* **7**, d244-d255 (2002).

9 Maisonpierre, P. C. *et al.* Angiopoietin-2, a natural antagonist for Tie2 that disrupts in vivo angiogenesis. *Science* **277**, 55-60 (1997).

10 Plank, M., Sleeman, B. & Jones, P. A mathematical model of tumour angiogenesis, regulated by vascular endothelial growth factor and the angiopoietins. *Journal of theoretical biology* **229**, 435-454 (2004).

11 Cai, Y., Xu, S., Wu, J. & Long, Q. Coupled modelling of tumour angiogenesis, tumour growth and blood perfusion. *Journal of Theoretical Biology* **279**, 90-101 (2011).

12 Buchwald, P. FEM-based oxygen consumption and cell viability models for avascular pancreatic islets. *Theoretical Biology and Medical Modelling* **6**, 5 (2009).

13 Cameron, M. A. & Davis, A. L. A Mathematical Model of Angiogenesis in Glioblastoma Multiforme. (2009).

14 Stéphanou, A., McDougall, S. R., Anderson, A. R. & Chaplain, M. A. Mathematical modelling of the influence of blood rheological properties upon adaptative tumour-induced angiogenesis. *Mathematical and Computer Modelling* **44**, 96-123 (2006).

15 Pries, A., Secomb, T. & Gaehtgens, P. Structural adaptation and stability of microvascular networks: theory and simulations. *American Journal of Physiology-Heart and Circulatory Physiology* **275**, H349-H360 (1998).

16 Stéphanou, A. *et al.* How tumour-induced vascular changes alter angiogenesis: insights from a computational model. *Journal of theoretical biology* **419**, 211-226 (2017).

17 Netti, P. A., Roberge, S., Boucher, Y., Baxter, L. T. & Jain, R. K. Effect of transvascular fluid exchange on pressure–flow relationship in tumors: a proposed mechanism for tumor blood flow heterogeneity. *Microvascular research* **52**, 27-46 (1996).

18 Cai, Y., Zhang, J. & Li, Z. Multi-scale mathematical modelling of tumour growth and microenvironments in anti-angiogenic therapy. *Biomedical engineering online* **15**, 155 (2016).

19 Cai, Y., Wu, J., Li, Z. & Long, Q. Mathematical modelling of a brain tumour initiation and early development: a coupled model of glioblastoma growth, pre-existing vessel co-option, angiogenesis and blood perfusion. *PloS one* **11**, e0150296 (2016).

20 Soltani, M. & Chen, P. Numerical modeling of interstitial fluid flow coupled with blood flow through a remodeled solid tumor microvascular network. *PloS one* **8**, e67025 (2013).

21 Zhao, G. *et al.* Numerical simulation of blood flow and interstitial fluid pressure in solid tumor microcirculation based on tumor-induced angiogenesis. *Acta Mechanica Sinica* **23**, 477-483 (2007).

22 Alarcón, T., Byrne, H. M. & Maini, P. K. A cellular automaton model for tumour growth in inhomogeneous environment. *Journal of theoretical biology* **225**, 257-274 (2003).

23 Pries, A. R. & Secomb, T. W. Microvascular blood viscosity in vivo and the endothelial surface layer. *American Journal of Physiology-Heart and Circulatory Physiology* **289**, H2657-H2664 (2005).

24 Boucher, Y., Leunig, M. & Jain, R. K. Tumor angiogenesis and interstitial hypertension. *Cancer Research* **56**, 4264-4266 (1996).

25 Lunt, S. J., Fyles, A., Hill, R. P. & Milosevic, M. Interstitial fluid pressure in tumors: therapeutic barrier and biomarker of angiogenesis. (2008).

26 Lesart, A.-C., Van Der Sanden, B., Hamard, L., Estève, F. & Stéphanou, A. On the importance of the submicrovascular network in a computational model of tumour growth. *Microvascular Research* **84**, 188-204 (2012).

27 Shamsi, M., Saghafian, M., Dejam, M. & Sanati-Nezhad, A. Mathematical Modeling of the Function of Warburg Effect in Tumor Microenvironment. *Scientific reports* **8**, 8903 (2018).

28 Kashkooli, F. M., Soltani, M., Rezaeian, M., Taatizadeh, E. & Hamedi, M.-H. Image-based spatio-temporal model of drug delivery in a heterogeneous vasculature of a solid tumor—Computational approach. *Microvascular research* (2019).

29 Voutouri, C. *et al.* Experimental and computational analyses reveal dynamics of tumor vessel cooption and optimal treatment strategies. *Proceedings of the National Academy of Sciences*, 201818322 (2019).

30 Xu, J., Vilanova, G. & Gomez, H. A mathematical model coupling tumor growth and angiogenesis. *PloS one* **11**, e0149422 (2016).
